# New Polyketides from a Hydrothermal Vent Sediment Fungus *Trichoderma* sp. JWM29-10-1 and Their Antimicrobial Effects

**DOI:** 10.3390/md20110720

**Published:** 2022-11-16

**Authors:** Changrong Lai, Jiayi Chen, Jing Liu, Danmei Tian, Donghe Lan, Tongzheng Liu, Bin Wu, Hongkai Bi, Jinshan Tang

**Affiliations:** 1 Key Laboratory of Pharmacodynamic Constituents of Traditional Chinese Medicine and New Drug Research, International Cooperative Laboratory of Traditional Chinese Medicine Modernization and Innovative Drug Development of Ministry of Education (MOE) of China, Institute of Traditional Chinese Medicine and Natural Products, College of Pharmacy, Jinan University, Guangzhou 510632, China; 2 Helicobacter pylori Research Centre, Jiangsu Key Laboratory of Pathogen Biology, Department of Pathogen Biology, Nanjing Medical University, Nanjing 211166, China; 3 Ocean College, Zhejiang University, Zhoushan Campus, Zhoushan 316021, China

**Keywords:** marine-derived fungus, *Trichoderma* sp. JWM29-10-1, polyketide, antibacterial effect, antifungal effect

## Abstract

Marine fungi-derived secondary metabolites are still an important source for the discovery of potential antimicrobial agents. Here, five new polyketides (**1**, **2**, and **6**–**8**) and seven known compounds (**3**–**5** and **9**–**12**) were obtained from the culture of the marine-derived fungus *Trichoderma* sp. JWM29-10-1. Their structures were identified by extensive spectrographic data analyses, including 1D and 2D NMR, UV, IR, and HR-ESI-MS. Further, the absolute configurations of new compounds were determined by circular dichroism (CD) spectrum and alkali-hydrolysis in combination with the in situ dimolybdenum CD method. Subsequently, the antimicrobial effects of these isolated compounds were assessed by examining the minimal inhibition concentration (MIC) with the broth microdilution assay. Compounds **1** and **2** exhibited potent antimicrobial activity against *Helicobacter pylori*, including multidrug-resistant strains, with MIC range values of 2–8 µg/mL. Moreover, compound **1** showed significant inhibitory effects on the growth of Gram-positive pathogens, including methicillin-resistant *Staphylococcus aureus* (MRSA), *Enterococcus faecalis*, and vancomycin-resistant *Enterococcus faecium*, which greatly threaten human health. This study demonstrates that chromone derivatives **1**–**2**, especially for **1**, could be potential lead compounds for the development of new antimicrobial agents and provides insight for future medicinal chemistry research.

## 1. Introduction

Marine fungi have the ability to produce biologically active lead compounds due to their special living environment. Increasing numbers of marine natural products (MNP) from marine fungi have been newly discovered in recent years [1,2]. *Trichoderma* sp. fungus is a filamentous fungus that mainly exists in marine animals, plants, and sediments attached to the seafloor [3,4]. Numerous chemical and pharmacological investigations have proved that *Trichoderma* sp. strains can produce abundant secondary metabolites, which exert potential anti-phytopathogenic, insecticidal, cytotoxic, antibacterial, and antioxidant activities, etc. [4,5] Polyketides, an important type of secondary metabolites from *Trichoderma* sp., were reported with vital antibacterial and anti-phytopathogenic effects [6]. Khamthong et al. reported the isolation of two new polyketides from *Trichoderma aureoviride* PSU-F95 and discovered one compound exhibited a certain antibacterial activity against *Methicillin-resistant Staphylococcus aureus* (MRSA) [6]. As infectious diseases seriously threaten human health due to the frequent occurrence of antibiotic resistance [7], polyketides have attracted significant attention due to their diverse biological effects, especially for their remarkable antimicrobial effects [8].

As part of our ongoing search for antimicrobial bioactive compounds from marine fungi, twelve polyketides, including five new compounds (**1**–**2** and **6**–**8**) and seven known ones (**3**–**5** and **9**–**12**), were isolated from the ethyl acetate (EtOAc) extract of the culture of fungus *Trichoderma* sp. JWM29-10-1. Here we described the isolation and chemical characterization of these isolated compounds. Meantime, the antimicrobial effects of these compounds were evaluated, and their preliminary structure-activity relationship (SAR) was also discussed.

## 2. Results

**Figure 1:**
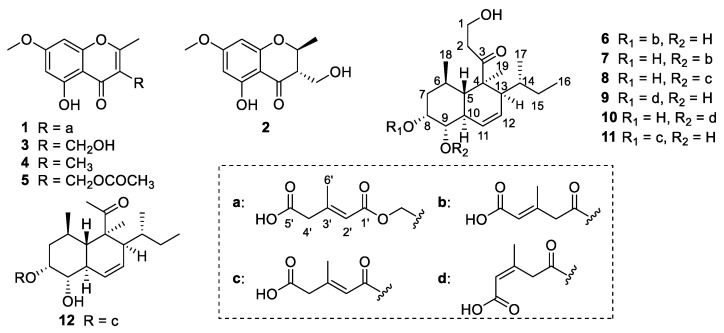
The chemical structures of compounds **1**–**12**.

Various chromatographic methods, including silica gel column chromatography (CC), ODS CC, and semi-preparative RP HPLC, were used to investigate the chemical components in *Trichoderma* sp. JWM29-10-1, which resulted in the isolation of five new polyketides (**1**–**2** and **6**–**8**) and seven known ones (**3**–**5** and **9**–**12**) (Figure 1). The known compounds were identified as 5-hydroxy-3-hydroxymethyl-2-methyl-7-methoxychromone (**3**) [9], 5-hydroxy-2,3-dimethyl-7-methoxychromone (**4**) [9], trichoharin A (**5**) [10], tandyukisin D (**9**) [11], tandyukisin C (**10**) [11], trichoharzin (**11**) [12], and deoxynortrichoharzin (**12**) [12] by comparing their MS, ^1^H and ^13^C NMR data, and specific rotation with those reported.

### 2.1. Structure Elucidation

**Figure 2:**
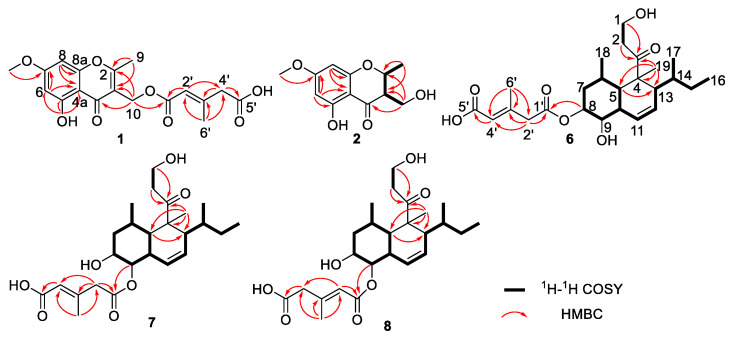
The key ^1^H-^1^H COSY and HMBC correlations of compounds **1**, **2**, and **6**–**8**.

Compound **1** was obtained as a colorless powder. It has the molecular formula of C_18_H_18_O_8_ with ten degrees of unsaturation by the HR-ESI-MS data at *m*/*z* 363.1084 [M + H]^+^ (calcd. *m*/*z* 363.1080, see Supplementary Materials). The UV absorptions at 208, 230, 250, 257, and 292 nm, and IR bands at 1677 cm^−1^ (conjugated carbonyl) and 1625, 1545 cm^−1^ (aromatic system) implicated the presence of a chromone core structure [9]. The ^1^H NMR spectrum showed resonances for phenolic hydroxyl at *δ*_H_ 12.63 (1H, s, 5-OH), two aromatic protons at *δ*_H_ 6.34 (2H, brs, H-6 and H-8), a methoxy at *δ*_H_ 3.85 (3H, s, 7-OMe), an oxymethylene at *δ*_H_ 5.11 (2H, s, H-10), a methylene at *δ*_H_ 3.15 (2H, s, H-4′), and two methyls at *δ*_H_ 2.50 (3H, s, H-9) and 2.25 (3H, s, H-6′) (Table 1). The ^13^C NMR spectrum aided by the DEPT135 spectrum revealed the presence of 18 carbon resonances for ten quaternary carbons, including three carbonyl carbons at *δ*_C_ 181.0 (C-4), 165.7 (C-1′), and 174.6 (C-5′), three oxygenated aromatic carbons at *δ*_C_ 167.6 (C-2), 162.3 (C-5), and 165.9 (C-7), and four aromatic or olefinic carbons; three methines at *δ*_C_ 98.2 (C-6), 92.6 (C-8), and 119.6 (C-2′); two methylenes at *δ*_C_ 56.0 (C-10) and 45.5 (C-4′); a methoxyl at *δ*_C_ 55.9 (7-OMe) and two methyls at *δ*_C_ 18.6 (C-9) and 19.2 (C-6′). A comparison of the NMR data with that reported revealed the core structure of **1** as 5-hydroxy-3-hydroxymethyl-2-methyl-7-methoxychromone [13]. Additionally, analyses of the ^1^H and ^13^C NMR data in combination with the HMBC correlations of H-6′ with C-2′–4′, of H-4′ with C-3′ and C-5′, and of H-2′ with C-1′ and C-4′ implicated the presence of a 3-methyl-2-pentenedioic acid side chain [14]. The HMBC correlation of H-10 with C-1′ attached the side chain at the hydroxymethyl of C-10. Thus, the planar structure of **1** was identified, which was confirmed by key HMBC correlations (Figure 2). Furthermore, the NOESY correlation of H-2′ with H-4′ indicated the *E* configuration of a double bond at C-2′ and C-3′. Thus, the structure of **1** was identified and named as (2*E*)-1-[(5-hydroxy-7-methoxy-2-methyl-4-oxo-4*H*-1-benzopyran-3-yl)methyl]3-methyl-2-pentenedioate.

**Figure 3:**
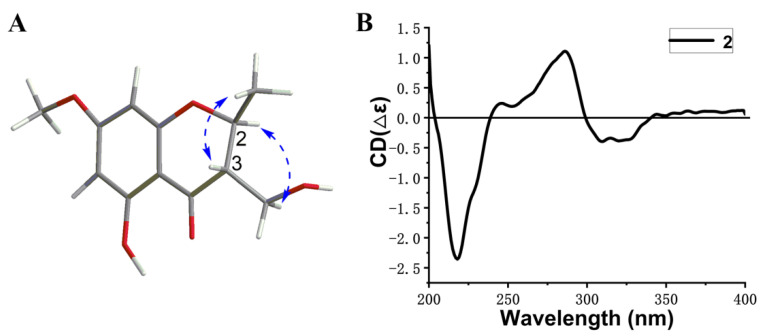
Key NOESY correlations (**A**) and CD spectrum (**B**) of **2**.

Compound **2** was isolated as a solid white powder. It has the molecular formula of C_12_H_14_O_5_ characterized by the HR-ESI-MS data at *m*/*z* 239.0925 [M + H]^+^ (calcd. *m*/*z* 239.0919), implying six degrees of unsaturation. Comparison of the ^1^H and ^13^C NMR data with that of 5,7-dihydroxy-2,3-dimethyl-4-chromanone suggested that **2** was also a chromone derivative [9], and the main difference laid in the ^13^C resonance of C-10. The ^13^C resonance of C-10 at 58.1 ppm in **2** instead of 9.7 ppm in 5,7-dihydroxy-2,3-dimethyl-4-chromanone indicated that **2** was the oxidative derivative of C-10 in 5,7-dihydroxy-2,3-dimethyl-4-chromanone [15]. Thus, the planar structure of **2** was identified, which was confirmed by ^1^H-^1^H COSY and HMBC correlations (Figure 2). The relative configuration of **2** was established by a NOESY experiment (Figure 3). In the NOESY spectrum, the correlations of H-3 with H-9 and of H-2 with H-10 indicated the *trans* orientation of H-2 and H-3, leading to the relative configuration of 2S* and 3S*. Since **2** showed opposite Cotton effects at 218 (∆ε, −2.4), 286 (∆ε, 1.1), and 310 (∆ε, −0.4) nm with those of (2*R*, 3*R*)-5,7-dihydroxy-2,3-dimethyl-4-chromanone [15], the absolute configuration of **2** was determined to be 2*S*, 3*S*. The opposite specific optical rotation of **2** with that of (2*R*, 3*R*)-5,7-dihydroxy-2,3-dimethyl-4-chromanone confirmed the above deduction [15]. Thus, the structure of **2** was determined and named (2*S*,3*S*)-5-hydroxy-3-hydroxymethyl-7-methoxy-2-methyl-4-chromanone.

**Figure 4:**
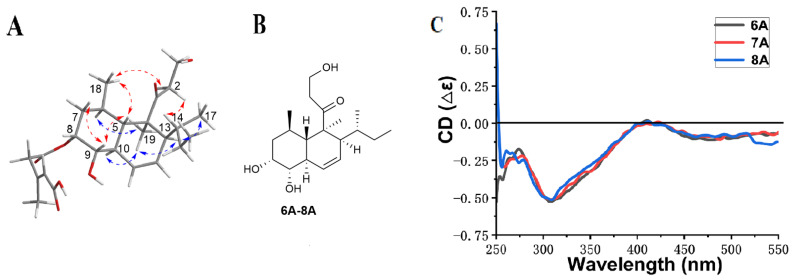
Key NOESY correlations of **6** (**A**), the hydrolytic products of **6**-**8**, **6A**-**8A** (**B**), and CD spectra of complexes of **6A**-**8A** with Mo_2_(OAc)_4_ (**C**).

Compound **6** was isolated as a pale-yellow oil, and its molecular formula was determined to be C_25_H_38_O_7_ by the HR-ESI-MS data at *m*/*z* 451.2707 [M + H]^+^ (calcd. 451.2696), accounting for seven degrees of unsaturation. Its IR spectrum exhibited characteristic bands at 3449, 1711, and 1623 cm^−1^ for hydroxyl groups, esters, and ketone, respectively. Analyses of the ^1^H and ^13^C NMR data aided by HSQC revealed five quaternary carbons, including three carbonyls at *δ*_C_ 215.5 (C-3), 170.2 (C-1′), and 170.4 (C-5′); ten methines including three olefinic methines at *δ*_H_ 6.01 (1H, brd, *J* = 10.4 Hz, H-11)/*δ*_C_ 125.7 (C-11), *δ*_H_ 5.70 (1H, brd, *J* = 10.4 Hz, H-12)/*δ*_C_ 124.1 (C-12), and *δ*_H_ 5.82 (1H, s, H-4′)/*δ*_C_ 119.3 (C-4′); two oxymethines at *δ*_H_ 5.24 (1H, d, *J* = 3.2 Hz, H-8)/*δ*_C_ 74.0 (C-8) and *δ*_H_ 3.56 (1H, dd, *J* = 10.8, 3.2 Hz, H-9)/*δ*_C_ 74.3 (C-9); five methylenes including one oxymethylene at *δ*_H_ 3.82 (1H, m, H-1b)/ 3.90 (1H, m, H-1a)/*δ*_C_ 58.1 (C-1), and five methyls (Table 2). A comparison of the NMR data with that of Tandyukisin D (**9**) implicated that they shared the same eujavanicol A core structure, and the main difference lay in the side chain. The side chain was identified to be (2*E*)-3-methyl-2-pentenedioic acid by the ^13^C NMR data analysis and key HMBC correlations (Figure 2). Further, the relative configuration of eujavanicol A fragment in **6** was identified by NOESY experiments. The NOESY correlations of H-19 with H-6, H-10, and H-13, and of H-13 with H-17 suggested that they were on one face (Figure 4). The NOESY correlations of H-5 with H-9 and H-18 indicated that they were on the other face and the decalin was *trans*. Thus, the relative configuration of **6** was identified as 4S*, 5S*, 6R*, 8R*, 9S*, 10R*, 13S*, 14R*, in line with that in **9**. Additionally, the ^13^C resonance of C-6′ at *δ*_C_ 19.4 ppm, less than 20 ppm, supported the *E* configuration of the double bond in **6** [16]. The absolute configuration of **6** was determined by chemical derivatization in combination with in situ dimolybdenum CD method. Treatment of **6** with NaOH aqueous in MeOH resulted in the acquisition of **6A**, which was identified to be eujavanicol A [17] according to their ^1^H and ^13^C NMR data and optical rotation value (**6A**: [α${]}_{D}^{25}$ +19.0, eujavanicol A: [α${]}_{D}^{25}$ +21.1), confirming the relative configuration of **6**. Subsequently, the CD spectrum of complexes formed by vic-diols in **6A** with dimolybdenum tetraacetate [Mo_2_(OAc)_4_] was measured. The negative Cotton effects at 310 and 400 nm arising within the d-d absorption bands of the metal complexes inferred the 8*R* and 9*S* configurations in **6A** (Figure 4) [18,19]. Thus, the absolute configuration of **6** was deduced as 4*S*, 5*S*, 6*R*, 8*R*, 9*S*, 10*R*, 13*S*, and 14*R*. Finally, the structure of **6** was identified and named Tandyukisin G.

Compound **7** was isolated as a pale-yellow oil. The HR-ESI-MS showed a quasimolecular ion at *m*/*z* 451.2699 [M + H]^+^ (calcd. 451.2696), indicating a molecular formula of C_25_H_38_O_7_ and accounting for seven degrees of unsaturation. Analyses of the ^1^H and ^13^C NMR data aided by HSQC, ^1^H-^1^H COSY, and HMBC revealed **7** was also a new decalin derivative containing a eujavanicol. A skeleton and a (2*E*)-3-methyl-2-pentenedioic acid side chain, the same as that of **6** (Figure 2). The HMBC correlation of H-9 with C-1′ attached the side chain at C-9 of the eujavanicol A fragment. In addition, the ^13^C resonance of C-6′ at *δ*_C_ 19.5 ppm deduced the *E* configuration of the double bond in the side chain [16]. The relative configuration of **7** was also determined by the NOESY experiment. Subsequently, alkali-hydrolysis of **7** produced **7A**, which was identified to be eujavanicol A by comparing their NMR data and optical rotation values with those reported [17]. Meanwhile, the negative Cotton effects of the complex formed by 8,9-diol in **7A** with Mo_2_(OAc)_4_ at 310 and 400 nm determined the 8*R*, 9*S* configurations of the eujavanicol A skeleton (Figure 4). Thus, the structure of **7** was unambiguously identified with the configurations of 4*S*, 5*S*, 6*R*, 8*R*, 9*S*, 10*R*, 13*S*, and 14*R* and named Tandyukisin H.

Compound **8** was obtained as a pale-yellow oil. Its molecular formula was elucidated as C_25_H_38_O_7_ based on the HR-ESI-MS (*m*/*z* 451.2697 [M + H]^+^, calcd. 451.2696) and ^13^C NMR data, implying seven degrees of unsaturation. A comparison of the ^1^H and ^13^C NMR data with that of **7** suggested that they had great similarity except for the side chain. The resonance signals of an olefinic methine at C-2′ [δ_H_ 5.89 (1H, s)/*δ*_C_ 119.4] and xa methylene at C-4′ [3.19 (2H, s)/*δ*_C_ 45.7] in **8** instead of methylene at δ_H_ 3.27 (2H, s)/*δ*_C_ 46.3 (C- 2′) and an olefinic methine at δ_H_ 5.85 (1H, s)/*δ*_C_ 119.5 (C-4′) in **7** suggested the presence of Δ^2′^ double bond in **8**. Meanwhile, the double bond was determined as an *E* configuration by the NOESY correlation of H-2′ with H-4′ in combination with the ^13^C resonance of C-6′ at *δ*_C_ 19.5 ppm [16]. The HMBC and ^1^H-^1^H COSY correlations confirmed the planar structure of **8** (Figure 2). The absolute configuration of 8,9-diols in **8** was also determined by alkali-hydrolysis followed by in situ dimolybdenum CD method. The negative Cotton effects of the complex formed by **8A** with Mo_2_(OAc)_4_ at 310 and 400 nm inferred the 8*R* and 9*S* configurations in **8**. Thus, the configurations of **8** were determined as 4*S*, 5*S*, 6*R*, 8*R*, 9*S*, 10*R*, 13*S*, and 14*R*. Finally, the structure of **8** was identified and named Tandyukisin I.

### 2.2. Antimicrobial Effects of Compounds **1**–**12**

**Figure 5:**
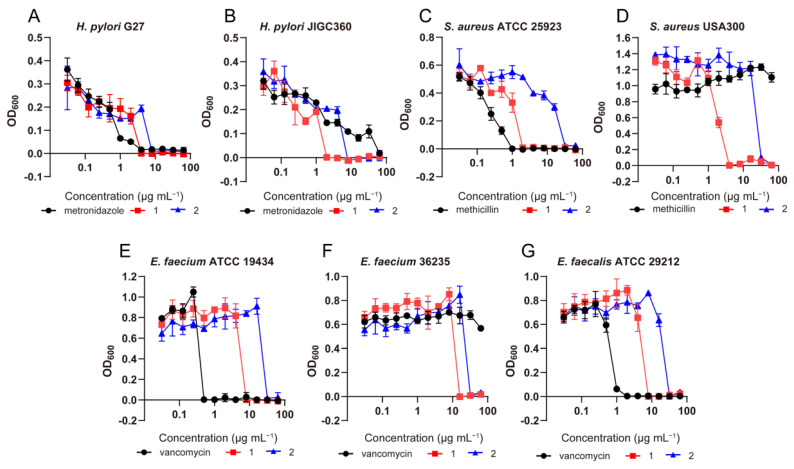
Growth of seven bacterial strains exposed to compounds **1** and **2** at various concentrations after 72 h for *H. pylori* strains and after 24 h for other bacterial strains. OD_600_, optical density at 600 nm. (**A**), Growth of *H. pylori* G27. (**B**), Growth of *H. pylori* JIGC360. (**C**), Growth of *S. aureus* ATCC 25923. (**D**), Growth of *S. aureus* USA300. (**E**), Growth of *E. faecium* ATCC 19434. (**F**), Growth of *E. faecium* 36235. (**G**), Growth of *E. faecalis* ATCC 29212.

The antimicrobial effects of compounds **1**–**12** were evaluated by the broth microdilution assay. Results showed that compounds **1** and **2** have efficient antibacterial activities against *Helicobacter pylori* standard strains and clinical isolates, including three multidrug-resistant strains, with minimal inhibition concentration (MIC) values ranging from 2–8 µg/mL (Table 3). Interestingly, compound **1** also exhibited significant inhibitory effects on the growth of Gram-positive pathogens, including *Staphylococcus aureus*, methicillin-resistant *S. aureus* (MRSA), vancomycin-resistant *Enterococcus faecium* (VRE) and *Enterococcus faecalis* with MIC values of 2 to 16 µg/mL (Table 3 and Figure 5). In addition, compound **1** exerted moderate antimicrobial activity against the important fungal pathogen *Candida albicans* and *Aspergillus fumigatus* with MIC values of 16 and 64 µg/mL, indicating that compound **1** has broad-spectrum antimicrobial activity. Preliminary structure-activity relationship (SAR) analysis revealed that the double bond at C-2 and C-3 of the chromone core structure might be unfavorable for its anti-*H. pylori* effects based on our limited results since compound **2** showed a stronger inhibitory effect than **3**. To our surprise, the introduction of a 3-methyl-2-pentenedioic acid side chain at C-13 could not only dramatically increase the anti-*H. pylori* activities of chromone derivatives, but also broad the antimicrobial spectrum from Gram-negative to Gram-positive bacteria and fungi.

## 3. Conclusions and Discussion

Marine fungal secondary metabolites have played a tremendous role in the discovery of anti-infectious drugs in the last 50 years [20,21,22]. In this study, five chromone derivatives (**1**–**5**) and seven decalin derivatives (**6**–**12**), including five new compounds (**1**, **2**, and **6**–**8**), were isolated from the culture of *Trichoderma* sp. JWM29-10-1. Chromone derivatives with 4-oxo-4*H*-1-benzopyran core scaffold represent a class of polyketides that were widely distributed in *Trichoderma* sp. and were reported with antifungal and cytotoxic effects. However, the systemic antimicrobial effect evaluation against bacteria and fungi and SAR were not performed up to now. Here compounds **1**–**2** exhibited potent antimicrobial effects with MIC values ranging from 2–16 µg/mL. Interestingly, compound **1** exhibited broad antimicrobial effects, especially for killing multidrug-resistant *H. pylori*. In addition, our study revealed that compound **1** showed significant inhibitory effects on the growth of MRSA and VRE, two of the major causes of community-acquired and hospital-acquired infections that threaten human health.

The rapid spread of multidrug-resistant (MDR) bacteria is a major concern for global public health. This threat is aggravated by an increasingly depleted antibiotic pipeline [23,24], with alarmingly few new classes of antibiotics introduced into clinical use over the past decades. In 2017, the World Health Organization (WHO) released a list of antibiotic-resistant “priority pathogens”—a catalogue of 12 bacterial species in urgent need of new antibiotics [25]. In this list, clarithromycin-resistant *H. pylori*, MRSA and VRE were ranked as ‘high” priority pathogens. In this study, compound **1** displayed a potent killing activity against these three important bacterial pathogens. To sum up, our studies shed light on the discovery of novel broad-spectrum antimicrobial agents and provide insight for future medicinal chemistry research.

## 4. Materials and Methods

### 4.1. General Experimental Procedure

Optical rotations were taken on a P-1020 digital polarimeter (JASCO International Co. Ltd., Tokyo, Japan). The IR spectra were measured on a JASCO FT/IR-480 plus spectrometer (JASCO, Tokyo, Japan), and UV/VIS spectra were recorded using a JASCO V-550 UV/VIS spectrometer (JASCO, Tokyo, Japan). Mass spectra were acquired with a Synapt G2 mass spectrometer (Waters, Wilmslow, UK). NMR data were taken by a Bruker AV 400 (Bruker Co. Ltd., Bremen, Germany) with signals of CD_3_OD (*δ*_H_ 3.31/*δ*_C_ 49.0) and CDCl_3_ (*δ*_H_ 7.26/*δ*_C_ 77.2) as internal references. A Chirascan plus (Applied Photo Physics Ltd., Leatherhead, UK) was used to acquire the CD spectra. The analytical and semi-preparative HPLC was carried out on a Shimadzu LC-20AB and LC-20AT Liquid Chromatography, respectively, with SPD-20A UV/VIS detector (Shimadzu, Tokyo, Japan). Columns for analytical and preparative HPLC were YMC-Triart C18 column (5 µm, ϕ 4.6 mm × 250 mm) and YMC Pack ODS-A column (5 µm, ϕ 10 mm × 250 mm), respectively. Silica gel for column chromatography (CC) was the product of Qingdao Marine Chemical Ltd. (Qingdao, China). ODS for CC were purchased from YMC Ltd. (YMC, Kyoto, Japan). MeOH and CH_3_CN with HPLC grade were purchased from Thermo Fisher (Waltham, MA, USA). Molybdenum acetate [Mo_2_(OAc)_4_] was purchased from Shanghai Yuanye Biological Co., Ltd (Shanghai, China).

The microorganism test strains were: Gram-negative bacteria (*H. pylori* strains G27, 159, JIGC360, and 511, *A. baumannii* ATCC 19,606, *E. coli* ATCC 25,922, and *P. aeruginosa* PAO1); Gram-positive bacteria (*S. aureus* ATCC 25,923, USA300, Newman, and NRS271, *E. faecium* ATCC 19,434, 36,235 and 36,711, *E. faecalis* ATCC 29,212); Fungus (*C. albicans* strains SC5314 and C5, *A. fumigatus* Af293). All were provided by the Department of Pathogen Biology & Jiangsu Key Laboratory of Pathogen Biology, Nanjing Medical University, Nanjing, China.

### 4.2. Fungal Material

The fungal strain JWM29-10-1 was collected and separated from hydrothermal vent sediments of Kueishantao, Taiwan, China and identified as *Trichoderma* sp. according to the morphological characteristics and the 18s rDNA sequence (OP501833), which is 99.9% similar to that of *Trichoderma reesei* (CBS999.97). The strain was preserved in Ocean College, Zhejiang University, Zhejiang, China.

### 4.3. Fermentation and Extraction

Strain *Trichoderma* sp. JWM29-10-1 was inoculated on a PDA agar plate, which consisted of 200 g potatoes, 20 g glucose, and 20 g agar in 1 L ddH_2_O. The spores from the agar plate were transferred into a triangular flask containing 100 mL PDA liquid medium and put in a constant temperature shaking incubator for 5 days (28 °C, 180 rpm/min) to obtain 1000 mL of seed culture solution (100 mL × 10). Then 20 mL of seed culture solution was inoculated to a solid rice medium composed of 100 g rice in 150 mL ddH_2_O. A total of 2.5 kg of large-scale fermentation was executed in a solid rice medium and cultured at room temperature for 45 days. The fermentation broth was extracted with EtOAc, and the filtrate was concentrated to dryness under reduced pressure to get crude extracts (30.0 g).

### 4.4. Compound Isolation

The crude extracts were chromatographed by silica gel CC (ϕ 8.0 *×* 50.0 cm, 200–300 mu, 400 g) eluted with gradient Petroleum ether-EtOAc (100:0, 98:2, 95:5, 9:1, 8:2, 7:3, 6:4, and 0:100) and EtOAc-MeOH (9:1) to obtain 6 fractions (Fr.1–6) based on TLC analyses.

The Fr.2 (1.25 g, Petroleum ether-EtOAc 80:20) was subjected to an ODS CC eluted with gradient MeOH–H_2_O (25–100%) to get 13 subfractions (Fr.2–1 to Fr.2–13). Fr.2–5 (39.2 mg, 45% MeOH–H_2_O) was purified by semi-preparative RP HPLC with an eluent of 60% MeOH–H_2_O (0.1% HCOOH) to get compound **2** (2.8 mg). Fr.2–6 (19.1 mg, 55% MeOH–H_2_O) was applied to semi-preparative RP HPLC eluted with 65% MeOH–H_2_O (0.1% HCOOH) to obtain compound **5** (6.5 mg). Fr.3 (2.06 g, Petroleum ether-EtOAc 70:30) was subjected to an ODS CC with stepwise gradient elution of 25%–100% MeOH–H_2_O to get 16 subfractions (Fr. 3–1–Fr. 3–16). Fr.3–7 (124.4 mg, 55% MeOH–H_2_O) was isolated by semi-preparative RP HPLC with an eluent of 65% MeOH–H_2_O (0.1%HCOOH) to yield compounds **3** (7.9 mg) and **4** (37.9 mg). Fr. 3–11 (74.1 mg, 65% MeOH-H_2_O) was applied to semi-preparative RP HPLC (70% MeOH-H_2_O with 0.1% HCOOH) to produce compounds **1** (3.4 mg), **11** (9.1 mg), and **12** (29.4 mg). Fr.4 (3.08 g, Petroleum ether-EtOAc 60:40) was applied to an ODS CC with gradient elution of 25–100% MeOH–H_2_O to get 13 subfractions (Fr. 4–1–Fr. 4–13). Fr.4–9 (224.4 mg, 65% MeOH–H_2_O) was purified by semi-preparative RP HPLC with an eluent of 35% CH_3_CN–H_2_O (0.1% HCOOH) to produce compounds **6** (28.4 mg), **7** (14.2 mg), **8** (22.7 mg), **9** (45.7 mg), and **10** (37.9 mg).

### 4.5. Spectroscopic Data of New Compounds

(2*E*)-1-[(5-hydroxy-7-methoxy-2-methyl-4-oxo-4*H*-1-benzopyran-3-yl)methyl]3-methyl-2-pentenedioate (**1**): White solid powder; UV (MeOH) *λ*_max_ (log ε): 208 (4.1), 230 (4.1), 250 (4.0), 257 (3.8), and 292 (3.5) nm; IR (KBr) *ν*_max_: 3437, 3263, 2953, 2930, 1677, 1625, 1545, 1475, 1453, 1387, and 1212 cm^−1^; HR-ESI-MS: *m*/*z* 363.1084 [M + H]^+^ (calcd for C_18_H_19_O_8_, *m*/*z* 363.1080); ^1^H and ^13^C NMR spectral data (Table 1).

(2*S*,3*S*)-5-hydroxy-3-hydroxymethyl-7-methoxy-2-methyl-4-chromanone (**2**): White solid powder; [α${]}_{D}^{25}$ −26.8 (*c* 0.5, in MeOH); UV (MeOH) *λ*_max_ (log ε): 215 (3.9), 229 (3.7), 287 (3.8) nm; IR (KBr) *ν*_max_: 3439, 2984, 2950, 1727, 1668, 1572, 1433, 1374, 1173, 1068, 927, and 854 cm^−1^; CD (MeOH) *λ*_max_ (∆ε): 218 (−2.4), 286 (1.1), 310 (−0.4); HR-ESI-MS: *m*/*z* 239.0925 [M + H]^+^ (calcd for C_12_H_15_O_5_, *m*/*z* 239.0919); ^1^H and ^13^C NMR spectral data (Table 1).

Tandyukisin G (**6**): Pale yellow oil; [α${]}_{D}^{25}$ +19.6 (*c* 0.5, in MeOH); UV (MeOH) *λ*_max_ (log ε): 215 (3.9) nm; IR (KBr) *ν*_max_: 3449, 2939, 1711, 1623, 1415, 1383, 1215, 1078, 988, and 926 cm^−1^; HR-ESI-MS: *m*/*z* 451.2707 [M + H]^+^ (calcd for *m*/*z* C_25_H_39_O_7_, 451.2696); ^1^H and ^13^C NMR spectral data (Table 2).

Tandyukisin H (**7**): Pale yellow oil; [α${]}_{D}^{25}$ +16.8 (*c* 0.5, in MeOH); UV (MeOH) *λ*_max_ (log ε): 214 (3.9) nm; IR (KBr) *ν*_max_: 3449, 2939, 1711, 1623, 1415, 1383, 1215, 1078, 988, and 926 cm^−1^; HR-ESI-MS: *m*/*z* 451.2699 [M + H]^+^ (calcd for C_25_H_39_O_7_, *m*/*z* 451.2696); ^1^H and ^13^C NMR spectral data (Table 2).

Tandyukisin I (**8**): Pale yellow oil; [α${]}_{D}^{25}$ −11.0 (*c* 0.5, in MeOH); UV (MeOH) λ_max_ (log ε): 220 (3.9) nm; IR (KBr) *ν*_max_: 3449, 2939, 1711, 1623, 1415, 1383, 1215, 1078, 988, and 926 cm^−1^; HR-ESI-MS: *m*/*z* 451.2697 [M + H]^+^ (calcd for C_25_H_39_O_7_, *m*/*z* 451.2696); ^1^H and ^13^C NMR spectral data (Table 2).

### 4.6. Absolute Configuration Determination of Compounds **6**–**8**

To a solution of compound **6** (16 mg) in 2.5 mL, MeOH was added, 2 mL of aqueous NaOH (1.0 M). Subsequently, the mixture was reacted at room temperature for 24 h. Then, the reaction mixture was extracted with MeOH thrice, and the organic layer was dried under reduced pressure to afford **6A** (11.5 mg). Following the same procedure, **7** (4.1 mg) and **8** (4.9 mg) were hydrolyzed with 0.3 M NaOH (aq.) to produce **7A** (2.9 mg) and **8A** (3.5 mg), respectively. Then, a mixture of **6A**, **7A**, or **8A** with dimolybdenum tetraacetate [Mo_2_(OAc)_4_] (1:1.2) in DMSO solution was kept to react for 30 min to form the chiral complexes. Then, the CD spectra of the complexes were measured.

### 4.7. Antimicrobial Assays

Antimicrobial assays were assessed by the broth microdilution assay following the previous literature [26,27,28] according to CLSI guidelines. Firstly, *H. pylori* strains were cultured in Brain Heart Infusion (BHI) medium containing 10% fetal calf serum (FCS) under microaerophilic conditions (85% N_2_, 10% CO_2_, 5% O_2_, and 90% relative humidity) using a double-gas CO_2_ incubator (Binder, model CB160; Tuttlingen, Germany), while other bacterial pathogens were aerobically cultured in Mueller-Hinton (MH) broth. *Candida albicans* and *Aspergillus fumigatus* strains were cultivated in Roswell Park Memorial Institute (RPMI) 1640 medium (Sigma, Kawasaki, Japan) containing L-glutamine and buffered with 165 mM MOPS at pH 7.0 (denoted as RPMI medium). Subsequently, a single colony was picked and continuously incubated in BHI/MH/RPMI broth to reach a logarithmic growth phase. Then, the test compounds were dissolved in DMSO and serially diluted two-fold to different concentrations on a 96-well plate. An aliquot (10 µL) of microbial suspension was added to each well, and cell concentration was adjusted to approximately 5 × 10^5^ cells/mL for *H. pylori*, 5 × 10^4^ cells/mL for other bacterial pathogens and 1 × 10^3^ cells/mL for fungi. The concentration range tested for each of the compounds was 64–0.125 µg/mL, and each compound was tested in triplicate. The negative control group was treated with sterile water. Metronidazole, methicillin, vancomycin, and amphotericin B were used as the positive control for *H. pylori*, *S. aureus*, and other bacteria and fungi, respectively. After incubating *H. pylori* at 37 °C for 72 h and other bacteria or fungi for 24 h, the plates were examined, and the MIC was defined as the lowest concentration of the compounds with no visible growth. Growth of seven bacterial strains exposed to compounds **1** and **2** at various concentrations after 72 h for *H. pylori* strains and after 24 h for other bacterial strains was examined at 600 nm for optical density, and the OD_600_ was recorded. Experiments were performed with three biological replicates.

## Figures and Tables

**Table 1:** ^1^H and ^13^C NMR data for compounds **1** and **2** (400 MHz for ^1^H and 100 MHz for ^13^C).

NO.	1 *^a^*		2 *^b^*	
	*δ*_C_	*δ*_H_ (*J* in Hz)	*δ*_C_	*δ*_H_ (*J* in Hz)
2	167.6		76.6	4.66 (1H, m)
3	114.8		54.3	2.59 (1H, dt, 9.6, 4.0)
4	181.0		198.2	
4a	104.9		104.1	
5	162.3		165.3	
6	98.2	6.34 (1H, brs)	95.5	6.02 (1H, d, 2.4)
7	165.9		169.5	
8	92.6	6.34 (1H, brs)	94.7	6.00 (1H, d, 2.4)
8a	157.6		164.0	
9	18.6	2.50 (3H, s)	19.3	1.54 (3H, d, 6.4)
10	56.0	5.11 (2H, s)	58.1	4.26 (1H, dd, 11.2, 4.0) 3.79 (1H, dd, 11.2, 4.0)
5-OH		12.63 (1H, s)		
7-OMe	55.9	3.85 (3H, s)	56.2	3.82 (3H, s)
1′	165.7			
2′	119.6	5.81 (1H, s)		
3′	151.5			
4′	45.5	3.15 (2H, s)		
5′	174.6			
6′	19.2	2.25 (3H, s)		

*^a^* Measured in CDCl_3_; *^b^* Measured in CD_3_OD.

**Table 2:** ^1^H and ^13^C NMR data for compounds **6**–**8** (400 MHz for ^1^H and 100 MHz for ^13^C in CDCl_3_).

NO.	6		7		8	
	*δ*_C_	*δ*_H_ (*J* in Hz)	*δ*_C_	*δ*_H_ (*J* in Hz)	*δ*_C_	*δ*_H_ (*J* in Hz)
1	58.1	3.90 (1H, m, Ha)	58.1	3.90 (1H, m, Ha)	58.1	3.93 (1H, m, Ha)
		3.82 (1H, m, Hb)		3.85 (1H, m, HB)		3.85 (1H, m, Hb)
2	41.3	2.85 (1H, brd,18.8, Ha)	41.3	2.88 (1H, brd, 18.8, Ha)	41.2	2.89 (1H, ddd, 18.8, 6.0, 3.6, Ha)
		2.66 (1H, brd, 18.8, Hb)		2.69 (1H, brd, 18.4, Ha)		2.70 (1H, ddd, 18.8, 7.2, 4.0, Ha)
3	215.5		215.3		215.5	
4	52.6		52.6		52.7	
5	43.2	1.96 (1H, m)	43.8	2.04 (1H, t, m)	43.7	2.05 (1H, m)
6	31.6	1.57 (1H, m)	30.4	1.80 (1H, m)	30.4	1.83 (1H, m)
7	39.0	1.82 (1H, brd,12.0, H*α*)	41.0	1.84 (1H, m, H*α*)	40.8	1.86 (1H, m, H*α*)
		1.55 (1H, m, H*β*)		1.58 (1H, brd, 13.6, H*β*)		1.58 (1H, m, H*β*)
8	74.0	5.24 (1H, d, 3.2)	67.6	4.15 (1H, brs)	67.7	4.15 (1H, q, 3.2)
9	74.3	3.56 (1H, dd, 10.8, 3.2)	79.1	4.75 (1H, d,11.2)	77.5	4.77 (1H, dd, 11.6, 2.8)
10	40.1	2.10 (1H, m)	36.3	2.46 (1H, t, 11.2)	36.3	2.50 (1H, m)
11	125.7	6.01 (1H, brd,10.4)	125.1	5.54 (1H, d, 10.8)	125.2	5.61 (1H, m)
12	124.1	5.70 (1H, brd,10.4)	124.6	5.69 (1H, d,10.8)	124.6	5.68 (1H, ddd, 10.4, 4.4,2.8)
13	52.4	1.93 (1H, m)	52.5	1.94 (1H, m)	52.5	1.94 (1H, m)
14	37.3	1.10 (1H, m)	37.3	1.11 (1H, m)	37.3	1.13 (1H, m)
15	24.6	0.74 (1H, m, a)	24.5	0.74 (1H, m)	24.5	0.73 (1H, m)
		1.45 (1H, m, b)		1.48 (1H, m)		1.49 (1H, m)
16	12.7	0.76 (3H, m)	12.6	0.77 (3H, t, 4.4)	12.7	0.76 (3H, t, 6.4)
17	19.4	0.92 (3H, d, 6.4)	19.3	0.92 (3H, d, 6.4)	19.3	0.91 (3H, d, 6.8)
18	22.4	0.58 (3H, d, 5.6)	22.4	0.60 (3H, d, 7.2)	22.4	0.60 (3H, d, 6.8)
19	19.5	1.23 (3H, s)	19.5	1.25 (3H, s)	19.4	1.25 (3H, s)
1′	170.2		169.0		165.1	
2′	46.4	3.23 (2H, s)	46.3	3.27 (2H, s)	119.4	5.89 (1H, s)
3′	153.7		153.4		152.5	
4′	119.3	5.82 (1H, s)	119.5	5.85 (1H, s)	45.7	3.19 (2H, s)
5′	170.4		170.3		174.3	
6′	19.4	2.24(3H, s)	19.5	2.27 (3H, s)	19.5	2.28 (3H, s)

**Table 3:** The antimicrobial effects of compounds **1**–**12** from the *Trichoderma* sp. JWM29-10-1 (MIC values, µg/mL).

			MIC (µg/mL) for:											
	Strain	Drug Sensitivity(Drug [s]) ^a^	1	2	3	4	5	6	7	8	9	10	11	12
Gram-Negative Bacteria	*Helicobacter pylori* G27	S	4	8	32	>64	32	>64	>64	>64	>64	>64	>64	64
	*Helicobacter pylori* 159	R (LVX, MTZ, CLR)	4	8	32	>64	32	>64	>64	>64	>64	>64	>64	64
	*Helicobacter pylori* JIGC360	R (LVX, MTZ)	2	8	16	>64	32	>64	>64	>64	>64	>64	>64	64
	*Helicobacter pylori* 511	R (LVX, MTZ, CLR)	2	8	16	>64	32	>64	>64	>64	>64	>64	>64	64
	*Acinetobacter baumannii* ATCC 19,606	S	>64	>64	>64	>64	>64	>64	>64	>64	>64	>64	>64	>64
	*Escherichia coli* ATCC 25,922	S	>64	>64	>64	>64	>64	>64	>64	>64	>64	>64	>64	>64
	*Pseudomonas aeruginosa* PAO1	S	>64	>64	>64	>64	>64	>64	>64	>64	>64	>64	>64	>64
Gram-Positive Bacteria	*Staphylococcus aureus* ATCC 25,923	S	2	32	>64	>64	>64	>64	>64	>64	>64	>64	>64	>64
	*Staphylococcus aureus* NEWMAN	S	4	32	>64	>64	>64	>64	>64	>64	>64	>64	>64	>64
	*Staphylococcus aureus* USA300	R (MET)	4	32	>64	>64	>64	>64	>64	>64	>64	>64	>64	>64
	*Staphylococcus aureus* NRS271	R (MET)	4	32	>64	>64	>64	>64	>64	>64	>64	>64	>64	>64
	*Enterococcus faecium* ATCC 19,434	S	8	32	>64	>64	>64	>64	>64	>64	>64	>64	>64	>64
	*Enterococcus faecium* 36,235	R (VAN)	16	32	>64	>64	>64	>64	>64	>64	>64	>64	>64	>64
	*Enterococcus faecium* 36,711	R (VAN)	16	32	>64	>64	>64	>64	>64	>64	>64	>64	>64	>64
	*Enterococcus faecalis* ATCC 29,212	S	8	32	>64	>64	>64	>64	>64	>64	>64	>64	>64	>64
Fungus	*Candida albicans* SC5314	S	16	>64	>64	>64	>64	>64	>64	>64	>64	>64	>64	>64
	*Candida albicans* C5	R (VOR, ITRA)	16	>64	>64	>64	>64	>64	>64	>64	>64	>64	>64	>64
	*Aspergillus fumigatus* Af293	S	64	>64	>64	>64	>64	>64	>64	>64	>64	>64	>64	>64

^a^ S, drug sensitive; R, drug resistant; LVX, levofloxacin; MTZ, metronidazole; CLR, clarithromycin; MET, methicillin; VAN, vancomycin; VOR, voriconazole; ITRA, itraconazole.

## Data Availability

The authors confirm that the data supporting the reported results are available within the article and its supplementary materials.

## Supplementary Materials

The following supporting information can be downloaded at: https://www.mdpi.com/article/10.3390/md20110720/s1, the HR-ESI-Q-TOF-MS, UV, IR, and 1D-, 2D-NMR spectra of compounds **1**–**2** (Figures S1–S19), **6**–**8** (Figures S20–S49), and **6A** (Figures S50–S52).
