# Supplementary material for: New Polyketides from a Hydrothermal Vent Sediment Fungus Trichoderma sp. JWM29-10-1 and Their Antimicrobial Effects
Source: Mar Drugs. 2022 Nov 16;20(11):720. doi: 10.3390/md20110720 (PMC9697660; doi:10.3390/md20110720)
Supplement: Supplementary file 1 [file marinedrugs-20-00720-s001.zip › marinedrugs-1997463-supplementary.pdf]

Supplementary **Information**

New polyketides from a hydrothermal vent sediment fungus *Trichoderma* sp. JWM29-10-1 and their antimicrobial effects

Changrong Lai [a, 1], Jiayi Chen [a, 1], Jing Liu [b, 1], Danmei Tian [a], Donghe Lan [c], Tongzheng Liu [a], Bin Wu [c, *], Hongkai Bi [b, *] and Jinshan Tang [a,*]

[a] *Institute of Traditional Chinese Medicine and Natural Products, College of Pharmacy/Guangdong Province Key Laboratory of Pharmacodynamic Constituents of Traditional Chinese Medicine and New Drug Research/International Cooperative Laboratory of Traditional Chinese Medicine Modernization and Innovative Drug Development of Ministry of Education (MOE) of China, Jinan University, Guangzhou 510632, China*

[b] *Department of Pathogen Biology, Jiangsu Key Laboratory of Pathogen Biology, Helicobacter pylori Research Centre, Nanjing Medical University, Nanjing 211166, China*

[c] *Ocean College, Zhejiang University, Zhoushan Campus, Zhoushan 316021, China*

[1] These authors contributed equally to this work.

Corresponding authors

Email address: jstang0615@jnu.edu.cn (J. Tang); hkbi@njmu.edu.cn (H. Bi); wubin@zju.edu.cn (B.W.)

Contents

Figure S1 HR-ESI-MS spectrum of compound **1** 1

Figure S2 UV spectrum of compound **1** 1

Figure S3 IR spectrum of compound **1** 2

Figure S4 ^{1}H-NMR spectrum of compound **1** (in $CDCl_3$) 2

Figure S5 ^{13}C-NMR spectrum of compound **1** (in $CDCl_3$) 3

Figure S6 DEPT135 spectrum of compound **1** (in $CDCl_3$) 3

Figure S7 HSQC spectrum of compound **1** (in $CDCl_3$) 4

Figure S8 HMBC spectrum of compound **1** (in $CDCl_3$) 4

Figure S9 NOESY spectrum of compound **1** (in $CDCl_3$) 5

Figure S10 HR-ESI-MS spectrum of compound **2** 5

Figure S11 UV spectrum of compound **2** 6

Figure S12 IR spectrum of compound **2** 6

Figure S13 ^{1}H-NMR spectrum of compound **2** (in CD_3OD) 7

Figure S14 ^{13}C-NMR spectrum of compound **2** (in CD_3OD) 7

Figure S15 DEPT135 spectrum of compound **2** (in CD_3OD) 8

Figure S16 ^{1}H-^{1}H COSY spectrum of compound **2** (in CD3OD) 8

Figure S17 HSQC spectrum of compound **2** (in CD_3OD) 9

Figure S18 HMBC spectrum of compound **2** (in CD_3OD) 9

Figure S19 NOESY spectrum of compound **2** (in CD_3OD) 10

Figure S20 HR-ESI-MS spectrum of compound **6** 10

Figure S21 UV spectrum of compound **6** 11

Figure S22 IR spectrum of compound **6** 11

Figure S23 ^{1}H-NMR spectrum of compound **6** (in $CDCl_3$) 12

Figure S24 ^{13}C-NMR spectrum of compound **6** (in $CDCl_3$) 12

Figure S25 DEPT135 spectrum of compound **6** (in $CDCl_3$) 13

Figure S26 ^{1}H-^{1}H COSY spectrum of compound **6** (in $CDCl_3$) 13

Figure S27 HSQC spectrum of compound **6** (in $CDCl_3$) 14

Figure S28 HMBC spectrum of compound **6** (in $CDCl_3$) 14

Figure S29 NOESY spectrum of compound **6** (in $CDCl_3$) 15

Figure S30 HR-ESI-MS spectrum of compound **7** 15
Figure S31 UV spectrum of compound **7** 16
Figure S32 IR spectrum of compound **7** 16
Figure S33 ^{1}H-NMR spectrum of compound **7** (in $CDCl_3$) 17
Figure S34 ^{13}C-NMR spectrum of compound **7** (in $CDCl_3$) 17
Figure S35 DEPT135 spectrum of compound **7** (in $CDCl_3$) 18
Figure S36 ^{1}H-^{1}H COSY spectrum of compound **7** (in $CDCl_3$) 18
Figure S37 HSQC spectrum of compound **7** (in $CDCl_3$) 19
Figure S38 HMBC spectrum of compound **7** (in $CDCl_3$) 19
Figure S39 NOESY spectrum of compound **7** (in $CDCl_3$) 20
Figure S40 HR-ESI-MS spectrum of compound **8** 20
Figure S41 UV spectrum of compound **8** 21
Figure S42 IR spectrum of compound **8** 21
Figure S43 ^{1}H-NMR spectrum of compound **8** (in $CDCl_3$) 22
Figure S44 ^{13}C-NMR spectrum of compound **8** (in $CDCl_3$) 22
Figure S45 DEPT135 spectrum of compound **8** (in $CDCl_3$) 23
Figure S46 ^{1}H-^{1}H COSY spectrum of compound **8** (in $CDCl_3$) 23
Figure S47 HSQC spectrum of compound **8** (in $CDCl_3$) 24
Figure S48 HMBC spectrum of compound **8** (in $CDCl_3$) 24
Figure S49 NOESY spectrum of compound **8** (in $CDCl_3$) 25
Figure S50 HR-ESI-MS spectrum of compound **6A** 25
Figure S51 ^{1}H-NMR spectrum of compound **6A** (in $CDCl_3$) 26
Figure S52 ^{13}C-NMR spectrum of compound **6A** (in $CDCl_3$) 26

Single Mass Analysis
Tolerance = 5.0 mDa / DBE: min = -1.5, max = 50.0
Element prediction: Off
Number of isotope peaks used for i-FIT = 3

Monoisotopic Mass, Even Electron Ions
46 formula(e) evaluated with 1 results within limits (up to 50 best isotopic matches for each mass)
Elements Used:
C: 0-30 H: 0-50 O: 0-10
JWM29-7-11-2
20220411-24 162 (1.313)

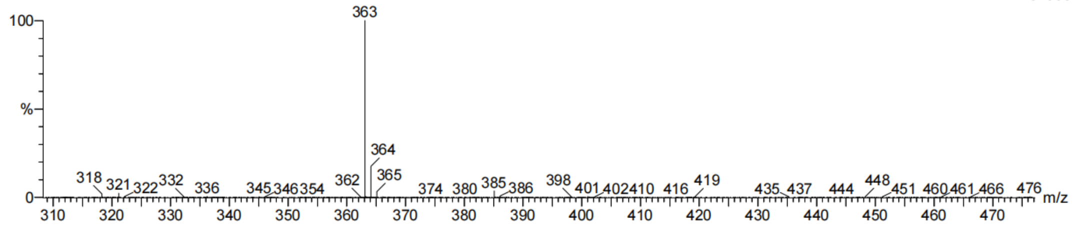

Minimum:				-1.5				
Maximum:		5.0	10.0	50.0				
Mass	Calc. Mass	mDa	PPM	DBE	i-FIT	Norm	Conf(%)	Formula
363.1084	363.1080	0.4	1.1	9.5	333.9	n/a	n/a	C18 H19 O8

Figure S1 HR-ESI-MS spectrum of compound **1**

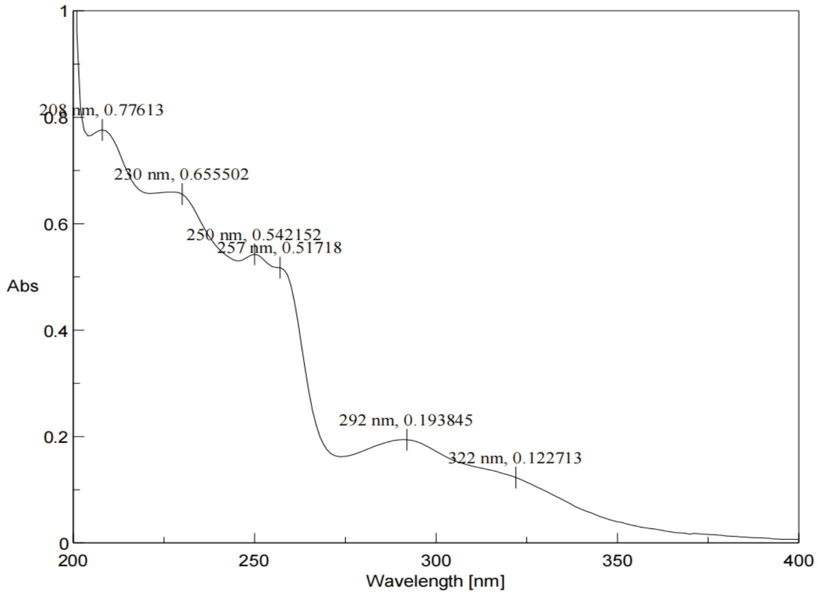

Figure S2 UV spectrum of compound **1**

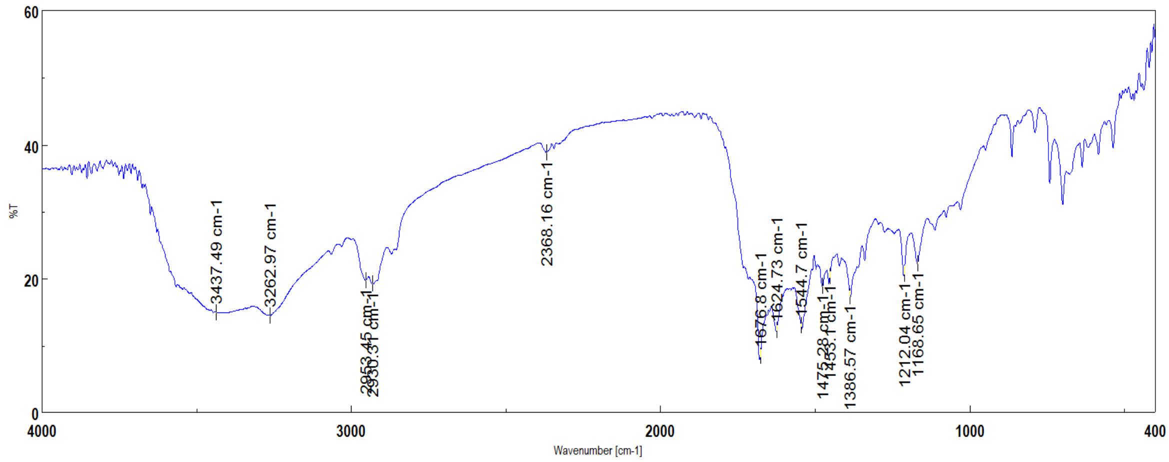

Figure S3 IR spectrum of compound **1**

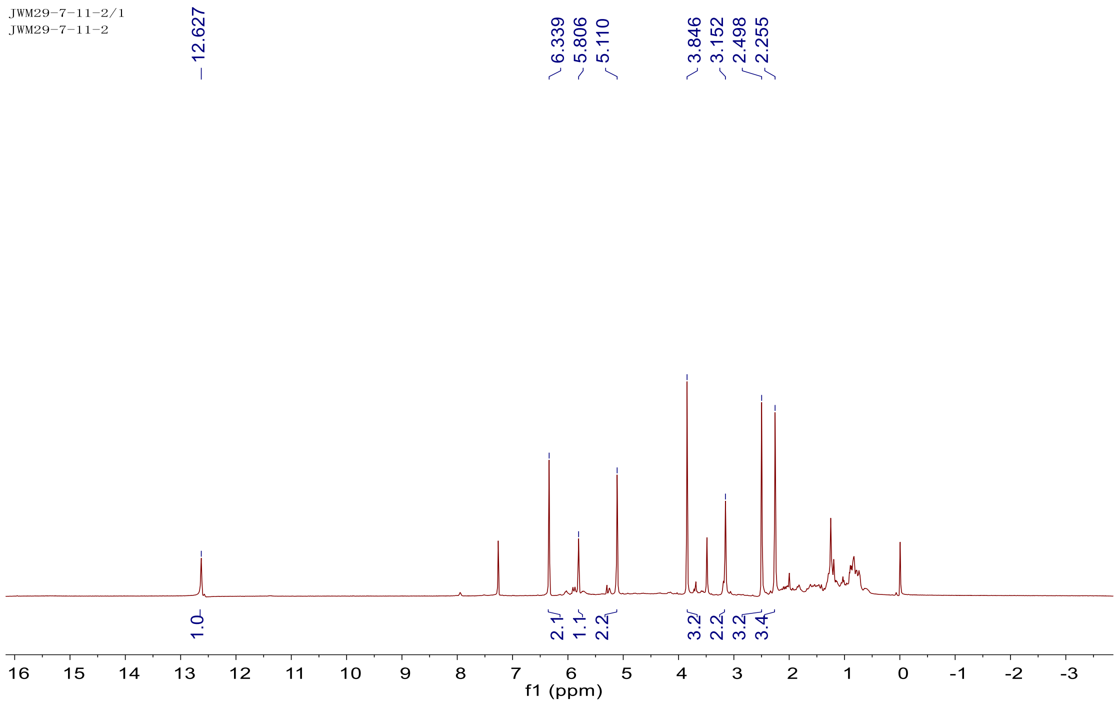

Figure S4 ^{1}H-NMR spectrum of compound **1** (in $CDCl_3$)

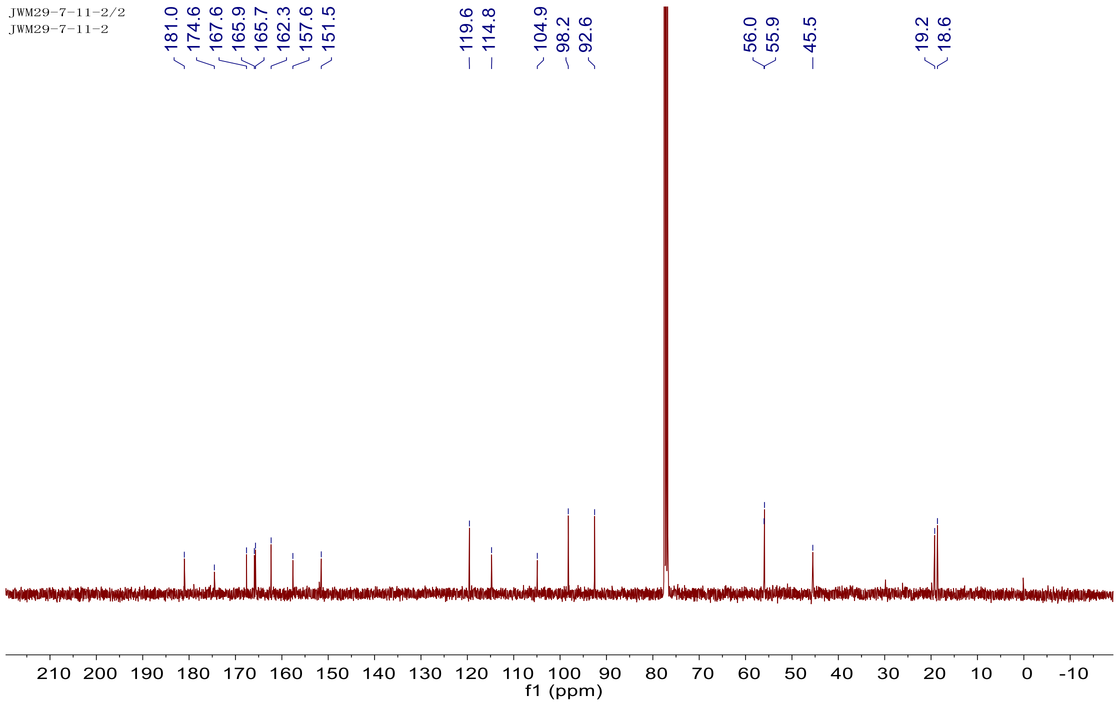

Figure S5 ^{13}C-NMR spectrum of compound **1** (in $CDCl_3$)

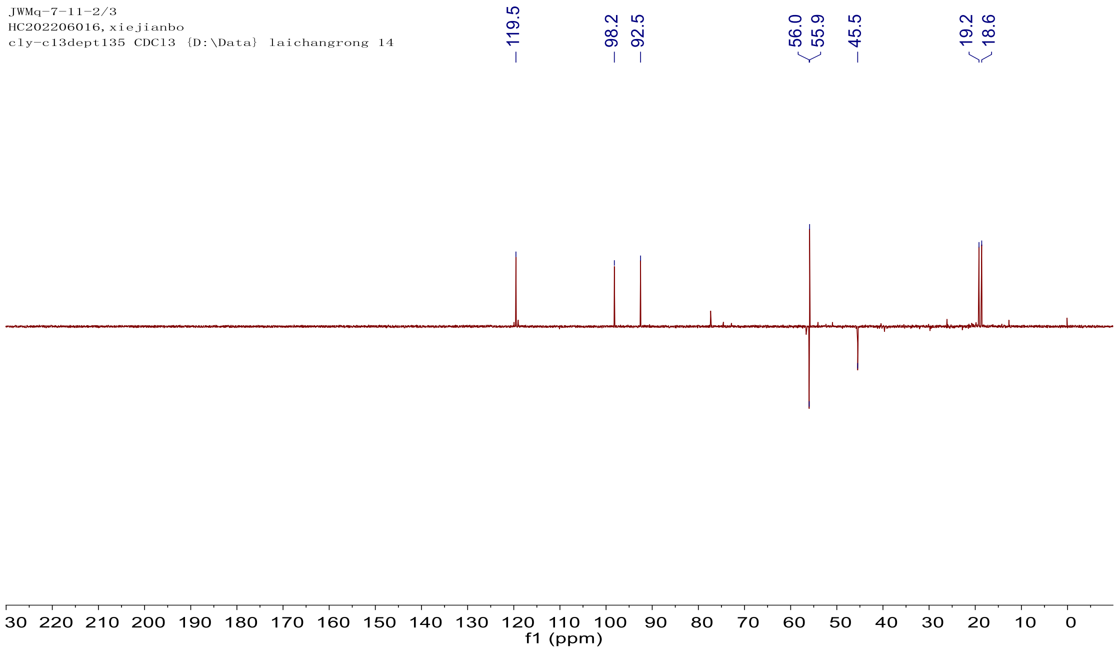

Figure S6 DEPT135 spectrum of compound **1** (in $CDCl_3$)

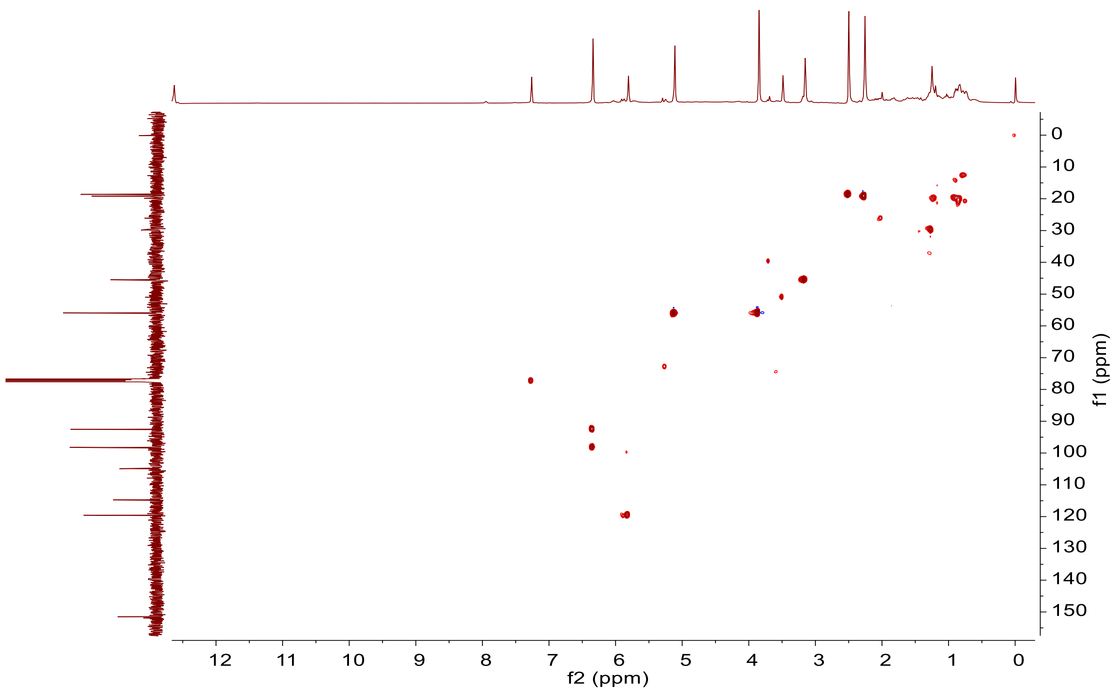

Figure S7 HSQC spectrum of compound **1** (in $CDCl_3$)

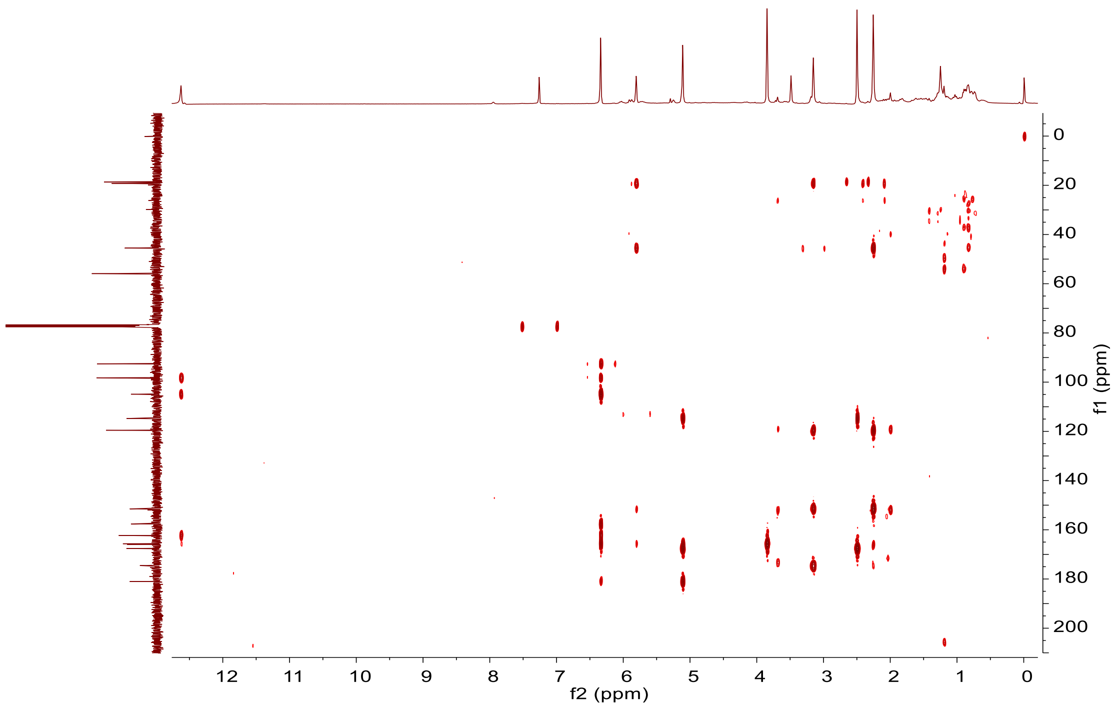

Figure S8 HMBC spectrum of compound **1** (in $CDCl_3$)

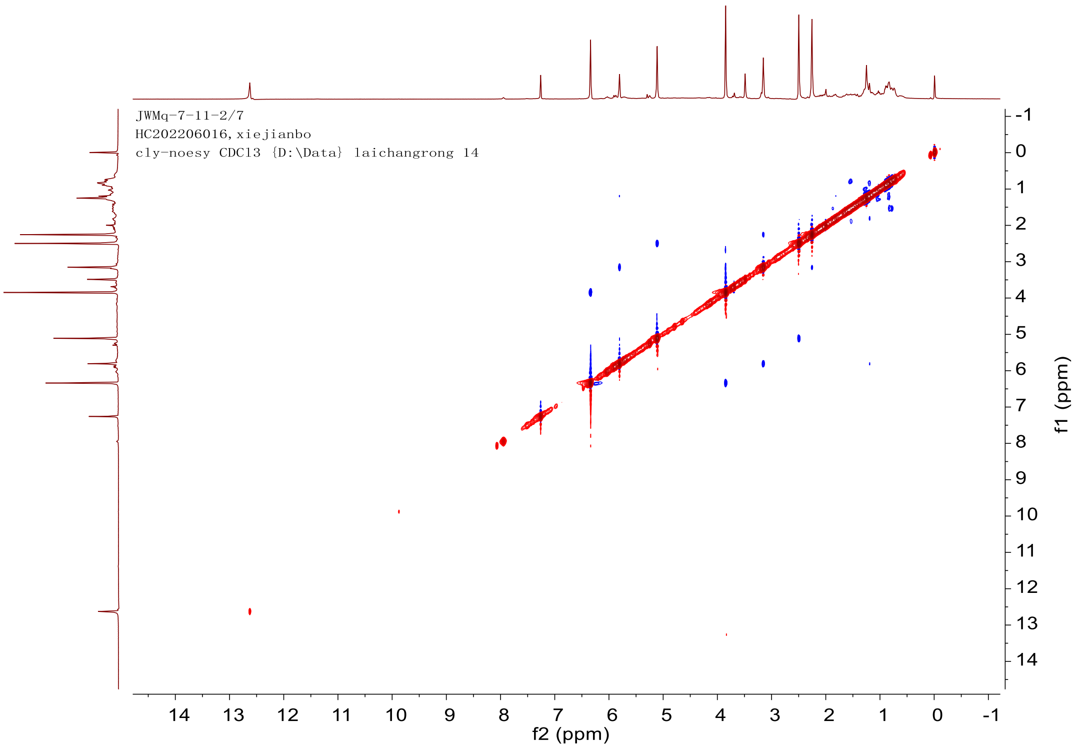

Figure S9 NOESY spectrum of compound **1** (in $CDCl_3$)

Single Mass Analysis
Tolerance = 5.0 mDa / DBE: min = -1.5, max = 50.0
Element prediction: Off
Number of isotope peaks used for i-FIT = 3

Monoisotopic Mass, Even Electron Ions
33 formula(e) evaluated with 1 results within limits (up to 50 closest results for each mass)
Elements Used:
C: 0-30 H: 0-50 O: 0-10
JWM29-3-5-4
20220322008 129 (1.044)

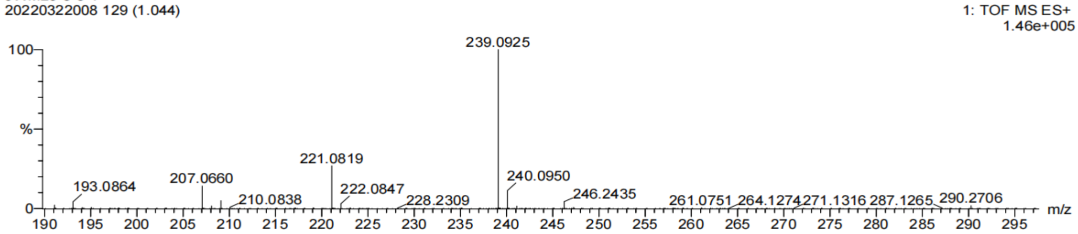

Minimum:				-1.5				
Maximum:		5.0	10.0	50.0				
Mass	Calc. Mass	mDa	PPM	DBE	i-FIT	Norm	Conf(%)	Formula
239.0925	239.0919	0.6	2.5	5.5	245.2	n/a	n/a	C12 H15 O5

Figure S10 HR-ESI-MS spectrum of compound **2**

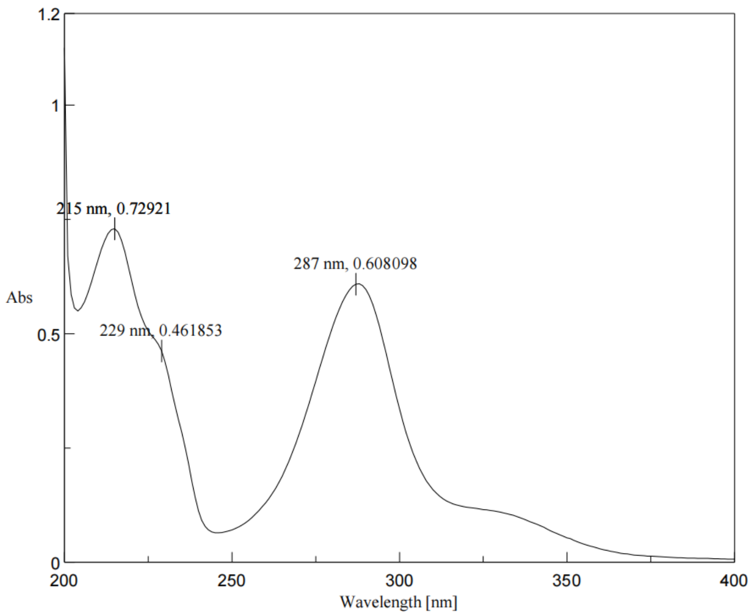

Figure S11 UV spectrum of compound **2**

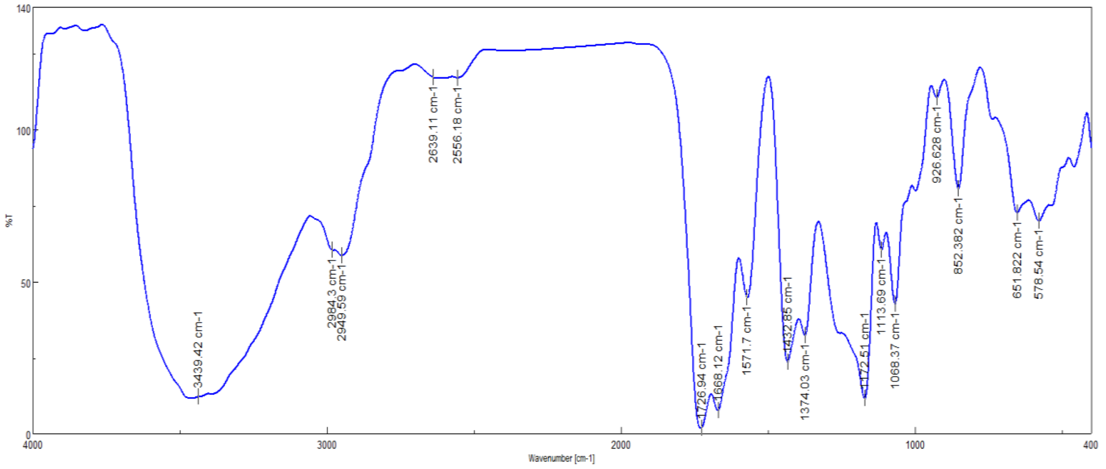

Figure S12 IR spectrum of compound **2**

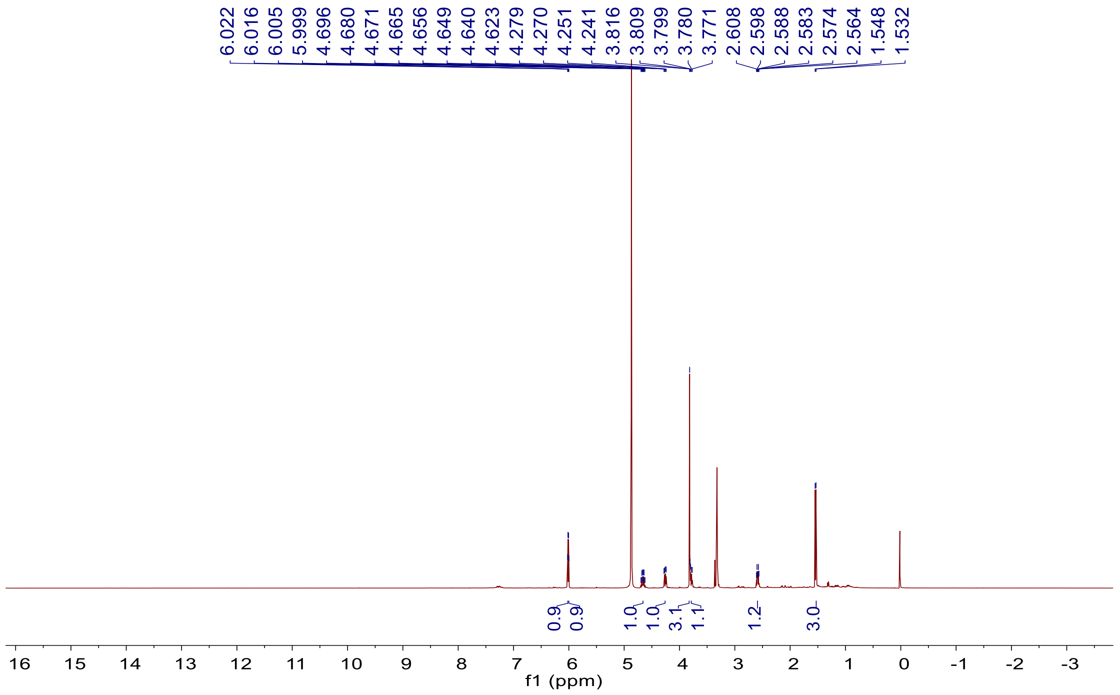

Figure S13 ^{1}H-NMR spectrum of compound **2** (in CD_3OD)

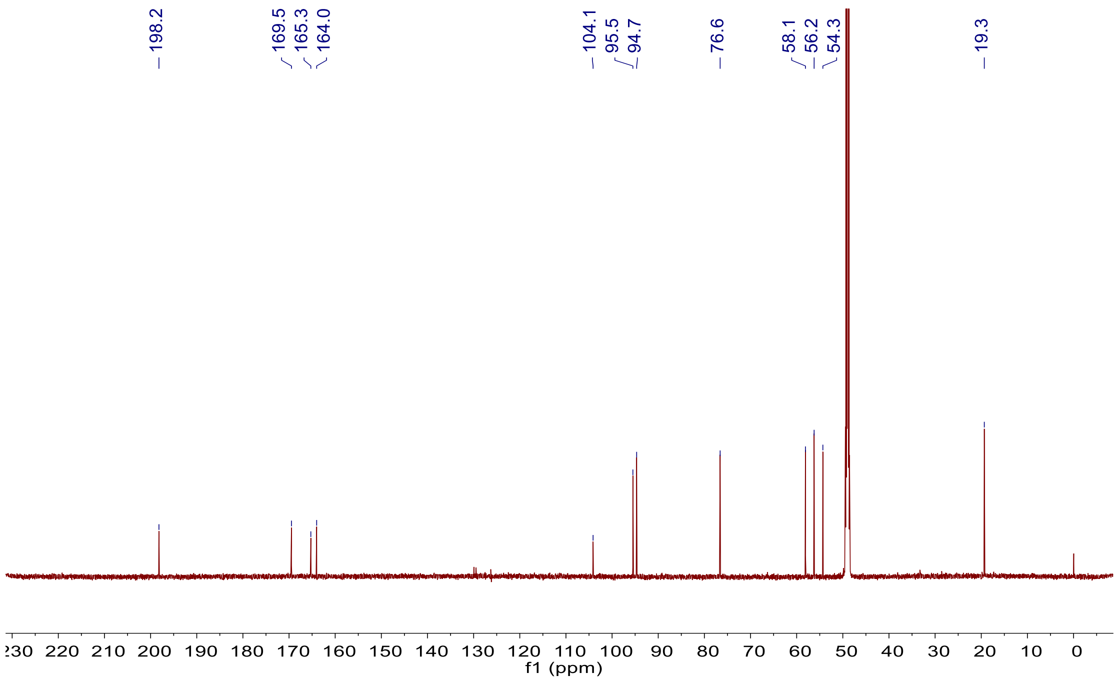

Figure S14 ^{13}C-NMR spectrum of compound **2** (in CD_3OD)

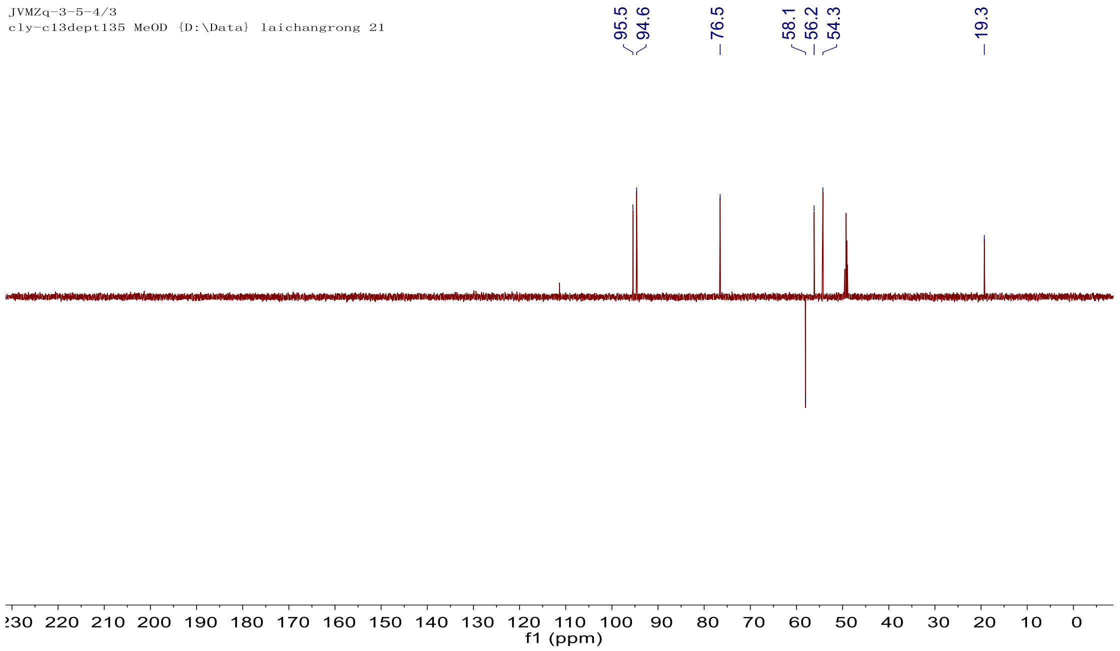

Figure S15 DEPT135 spectrum of compound **2** (in CD_3OD)

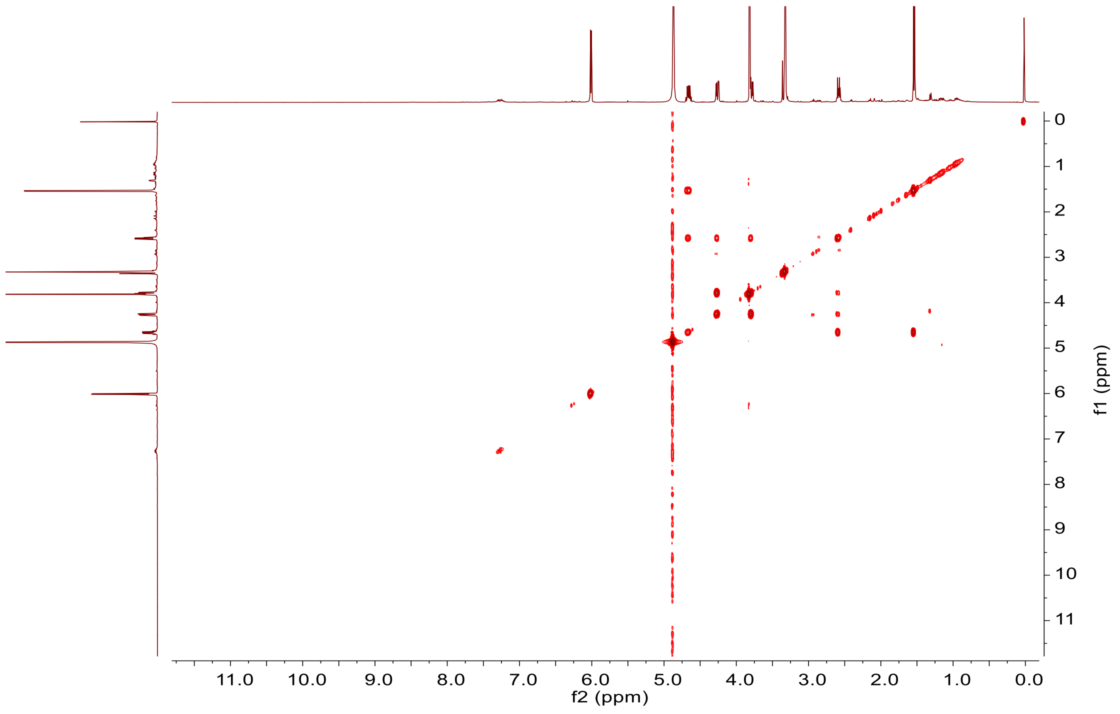

Figure S16 ^{1}H-^{1}H COSY spectrum of compound **2** (in CD_3OD)

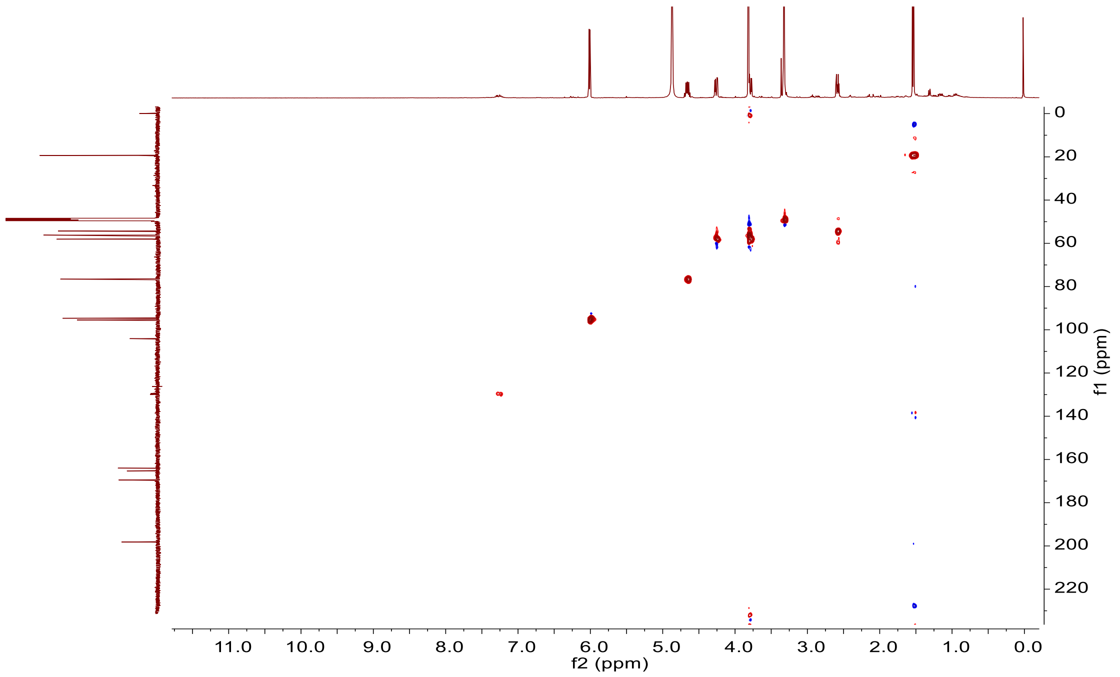

Figure S17 HSQC spectrum of compound **2** (in CD_3OD)

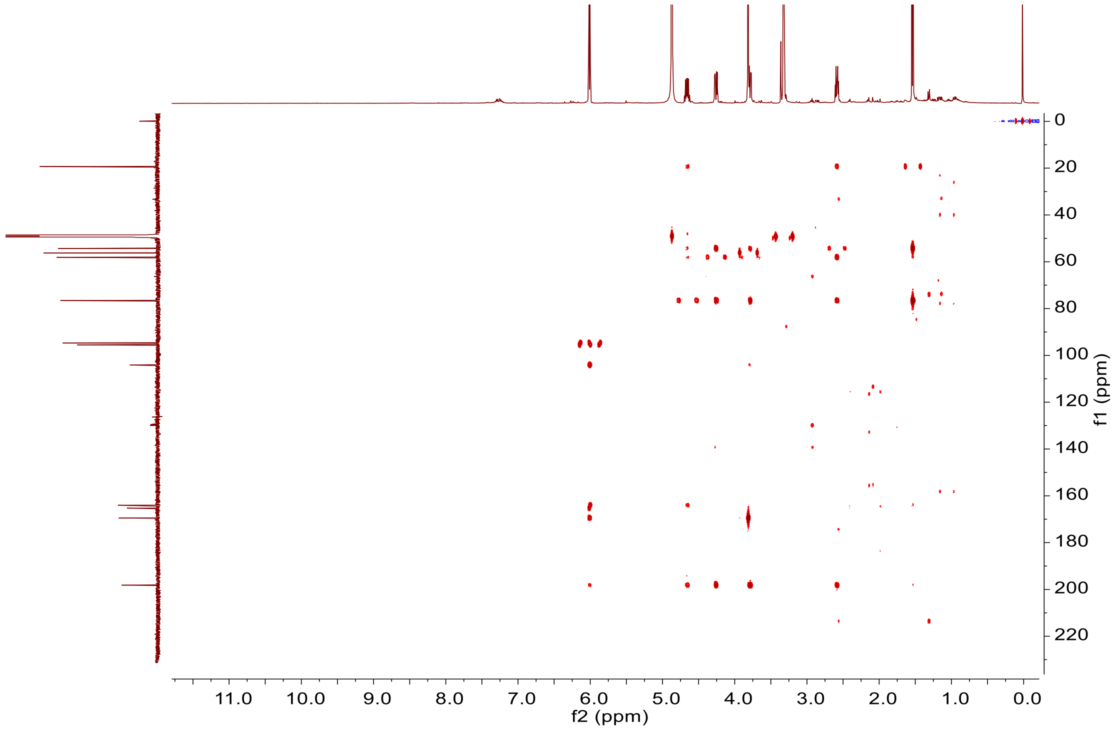

Figure S18 HMBC spectrum of compound **2** (in CD_3OD)

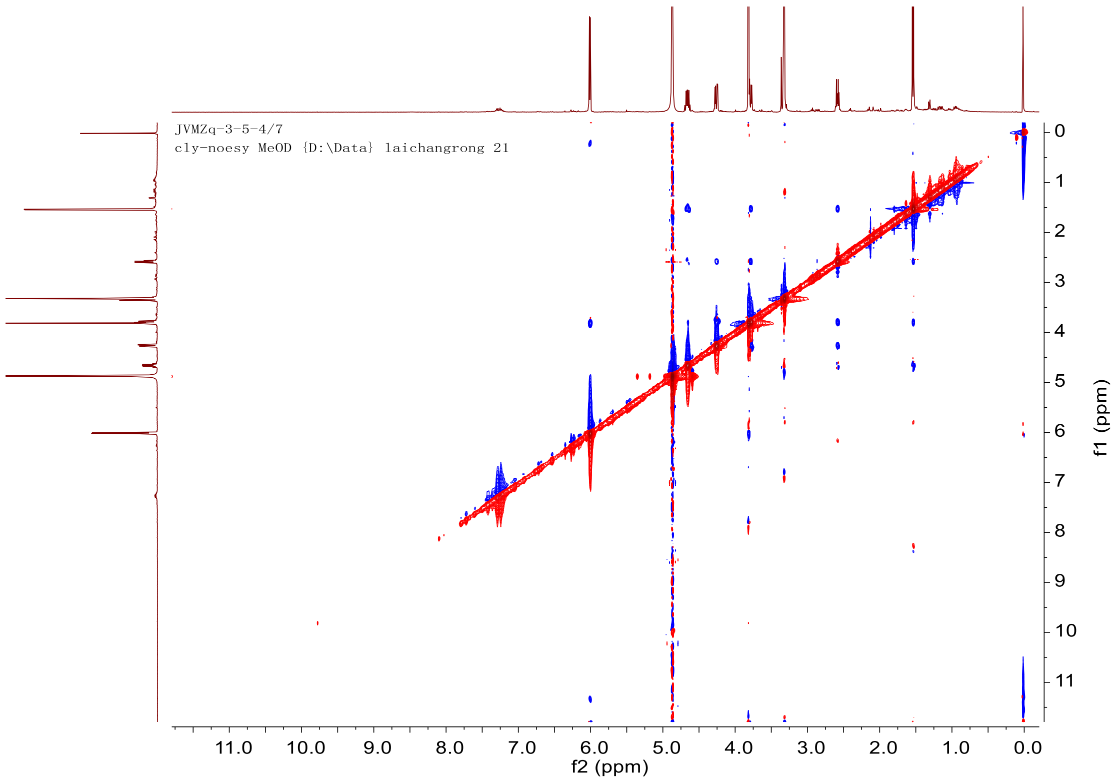

Figure S19 NOESY spectrum of compound **2** (in CD_3OD)

Single Mass Analysis
Tolerance = 5.0 mDa / DBE: min = -1.5, max = 50.0
Element prediction: Off
Number of isotope peaks used for i-FIT = 3

Monoisotopic Mass, Even Electron Ions
30 formula(e) evaluated with 1 results within limits (up to 50 best isotopic matches for each mass)
Elements Used:
C: 0-30 H: 0-50 O: 0-10
JWM29-9-9-1
20220613033 131 (1.070) 1: TOF MS ES+
9.00e+004

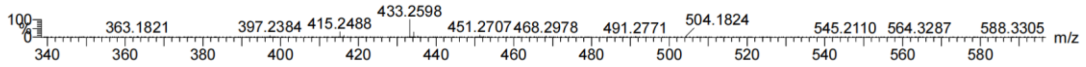

Minimum:				-1.5				
Maximum:		5.0	10.0	50.0				
Mass	Calc. Mass	mDa	PPM	DBE	i-FIT	Norm	Conf(%)	Formula
451.2707	451.2696	1.1	2.4	6.5	130.0	n/a	n/a	C25 H39 O7

Figure S20 HR-ESI-MS spectrum of compound **6**

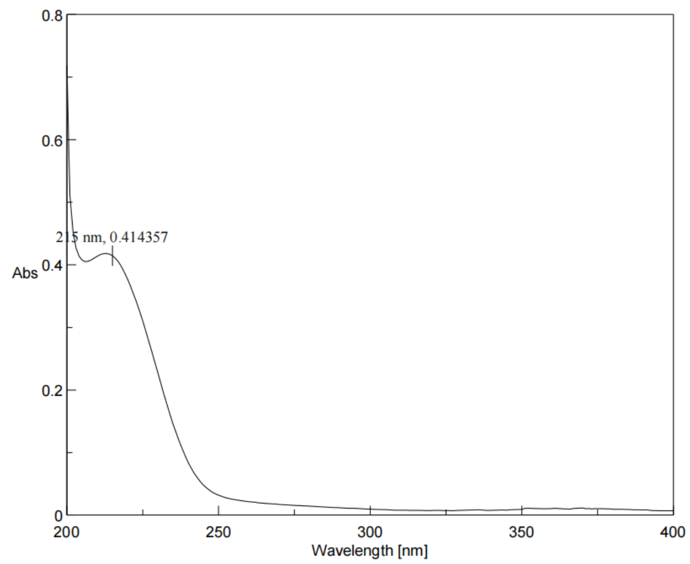

Figure S21 UV spectrum of compound **6**

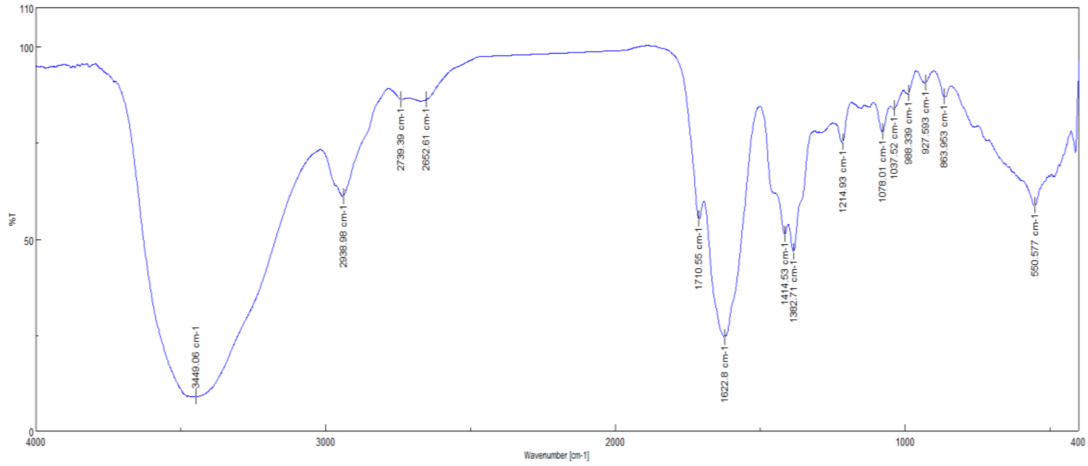

Figure S22 IR spectrum of compound **6**

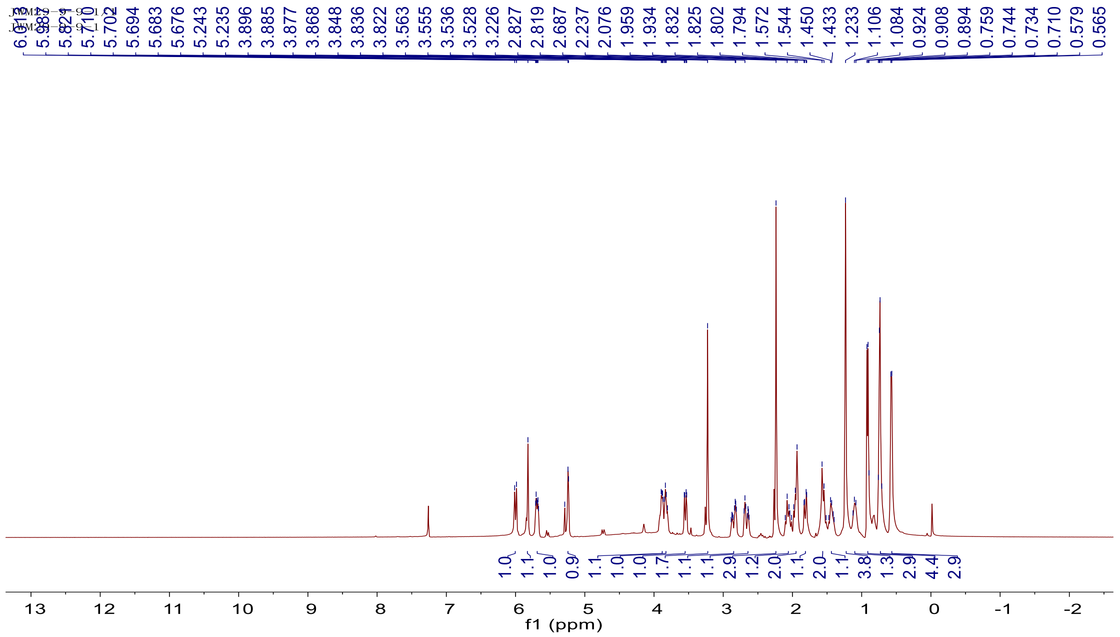

Figure S23 ^{1}H-NMR spectrum of compound **6** (in $CDCl_3$)

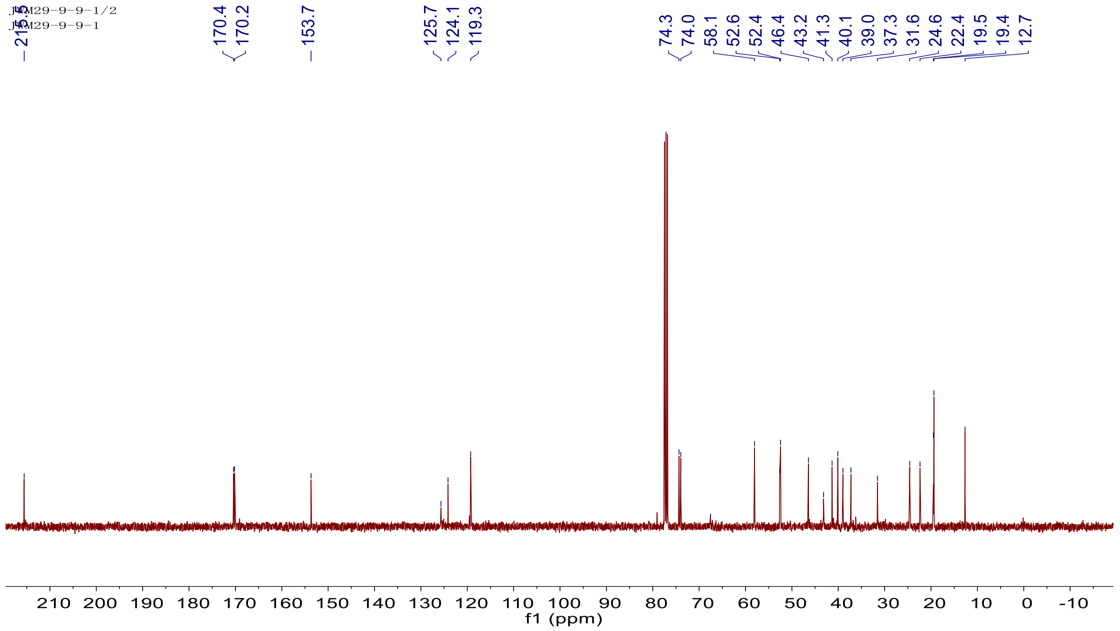

Figure S24 ^{13}C-NMR spectrum of compound **6** (in $CDCl_3$)

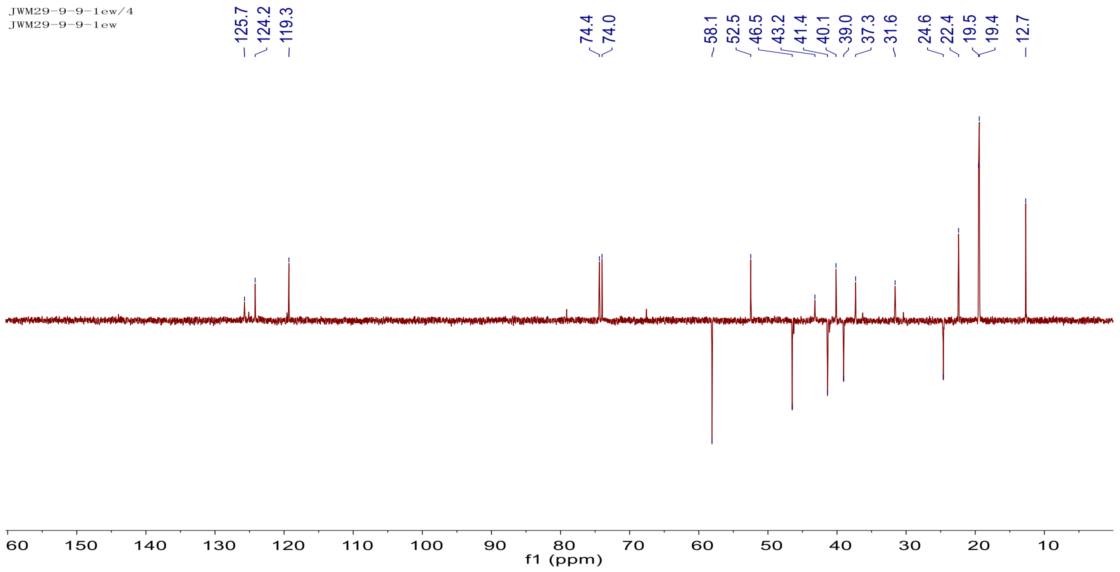

Figure S25 DEPT135 spectrum of compound **6** (in $CDCl_3$)

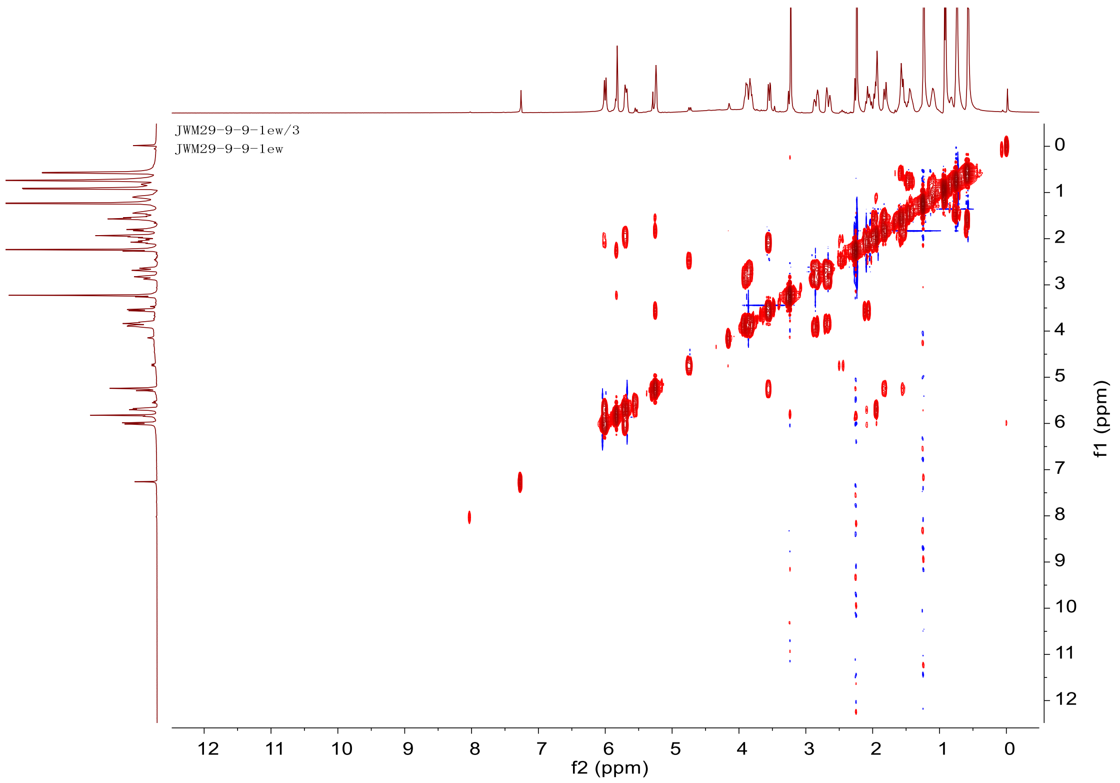

Figure S26 ^{1}H-^{1}H COSY spectrum of compound **6** (in $CDCl_3$)

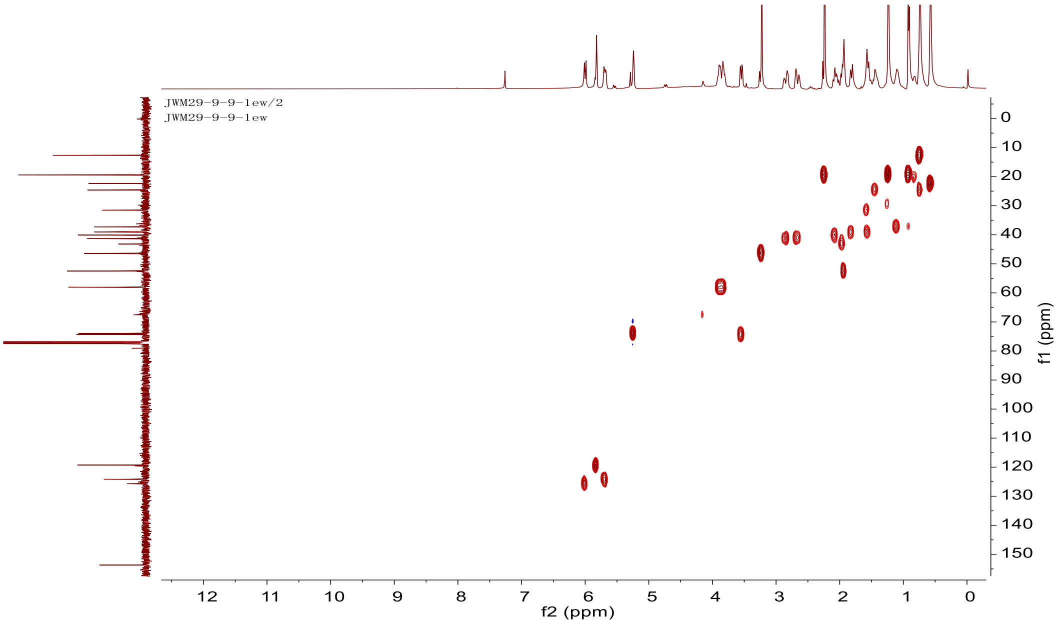

Figure S27 HSQC spectrum of compound **6** (in $CDCl_3$)

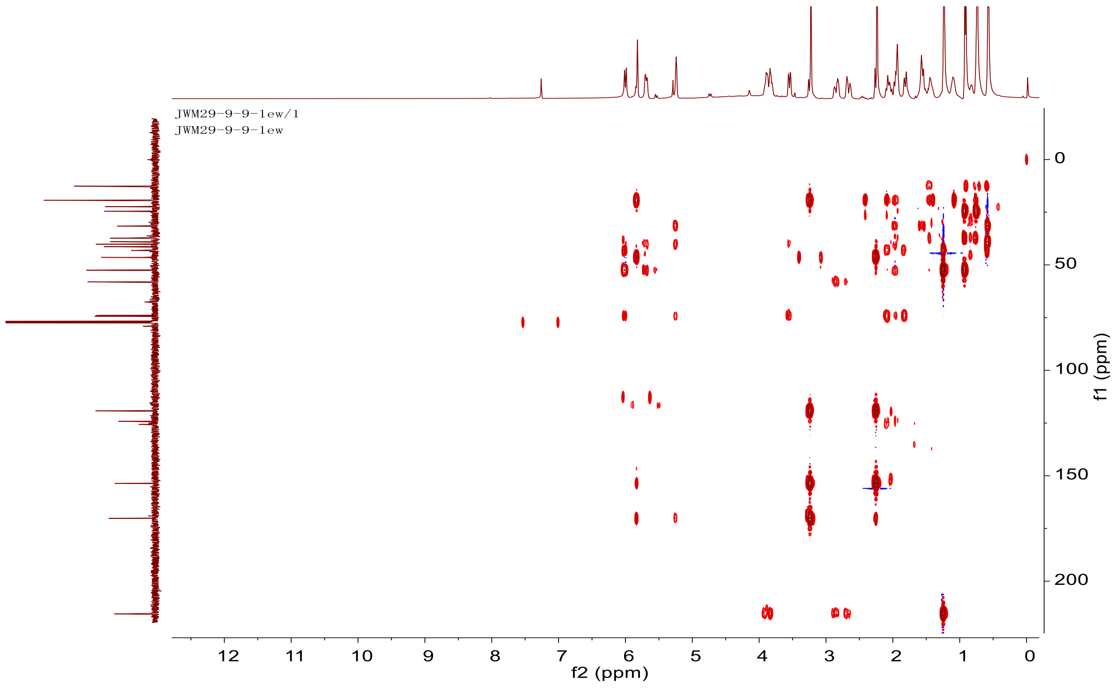

Figure S28 HMBC spectrum of compound **6** (in $CDCl_3$)

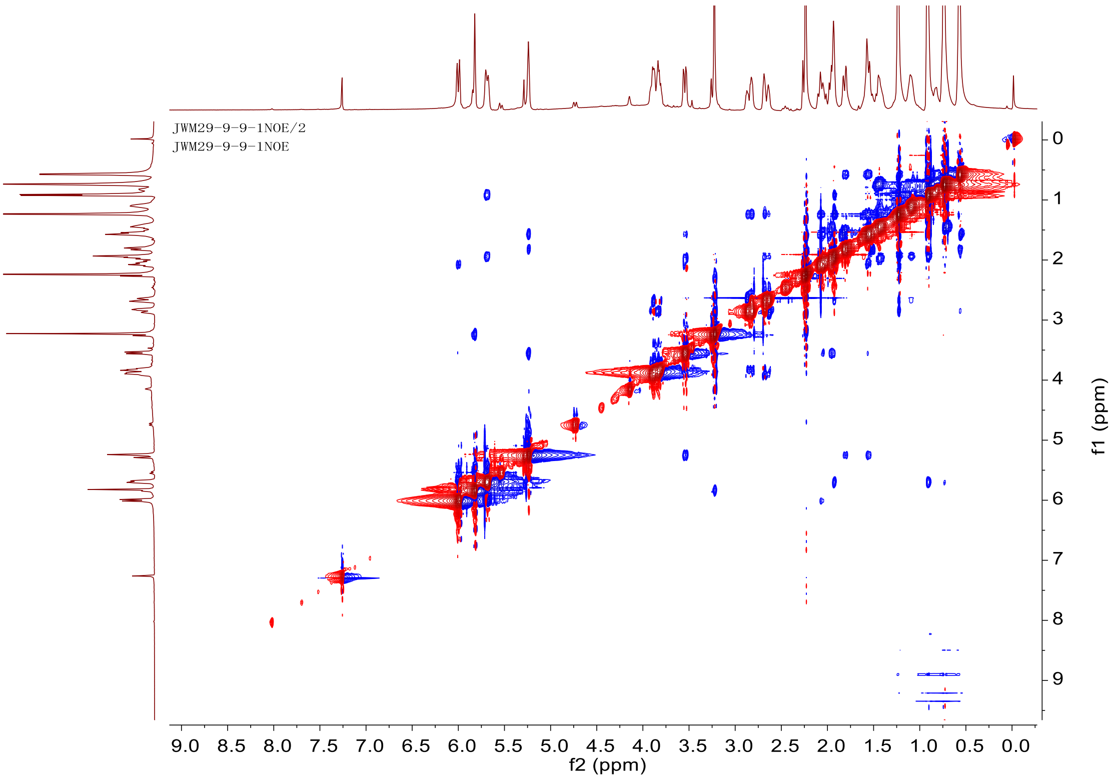

Figure S29 NOESY spectrum of compound **6** (in $CDCl_3$)

Single Mass Analysis
Tolerance = 5.0 mDa / DBE: min = -1.5, max = 50.0
Element prediction: Off
Number of isotope peaks used for i-FIT = 3

Monoisotopic Mass, Even Electron Ions
30 formula(e) evaluated with 1 results within limits (up to 50 best isotopic matches for each mass)
Elements Used:
C: 0-30 H: 0-50 O: 0-10
JWM29-9-9-2
20220613034 132 (1.077) 1: TOF MS ES+
3.73e+005

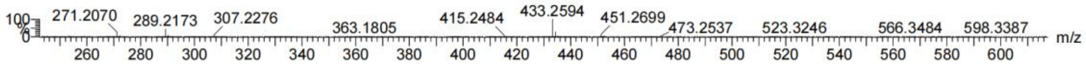

Minimum:				-1.5				
Maximum:		5.0	10.0	50.0				
Mass	Calc. Mass	mDa	PPM	DBE	i-FIT	Norm	Conf(%)	Formula
451.2699	451.2696	0.3	0.7	6.5	229.4	n/a	n/a	C25 H39 O7

Figure S30 HR-ESI-MS spectrum of compound **7**

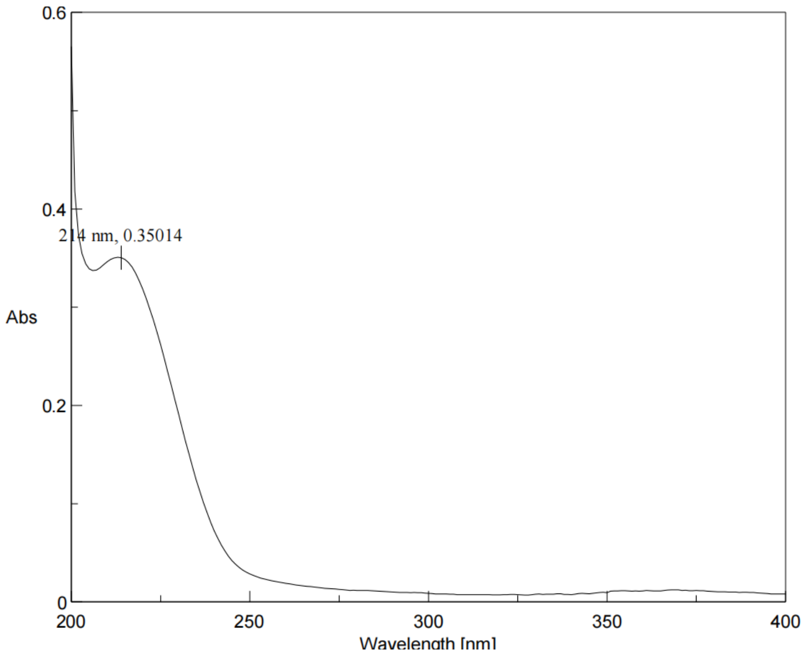

Figure S31 UV spectrum of compound **7**

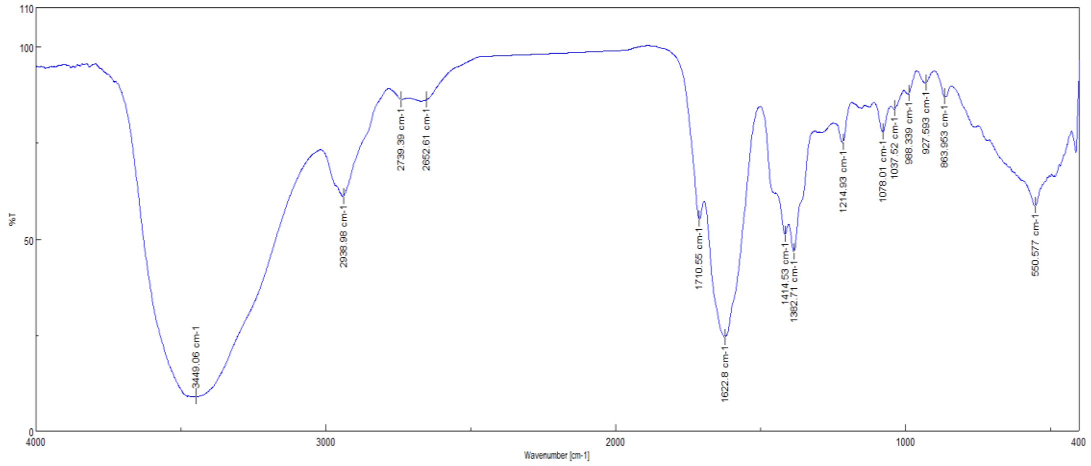

Figure S32 IR spectrum of compound **7**

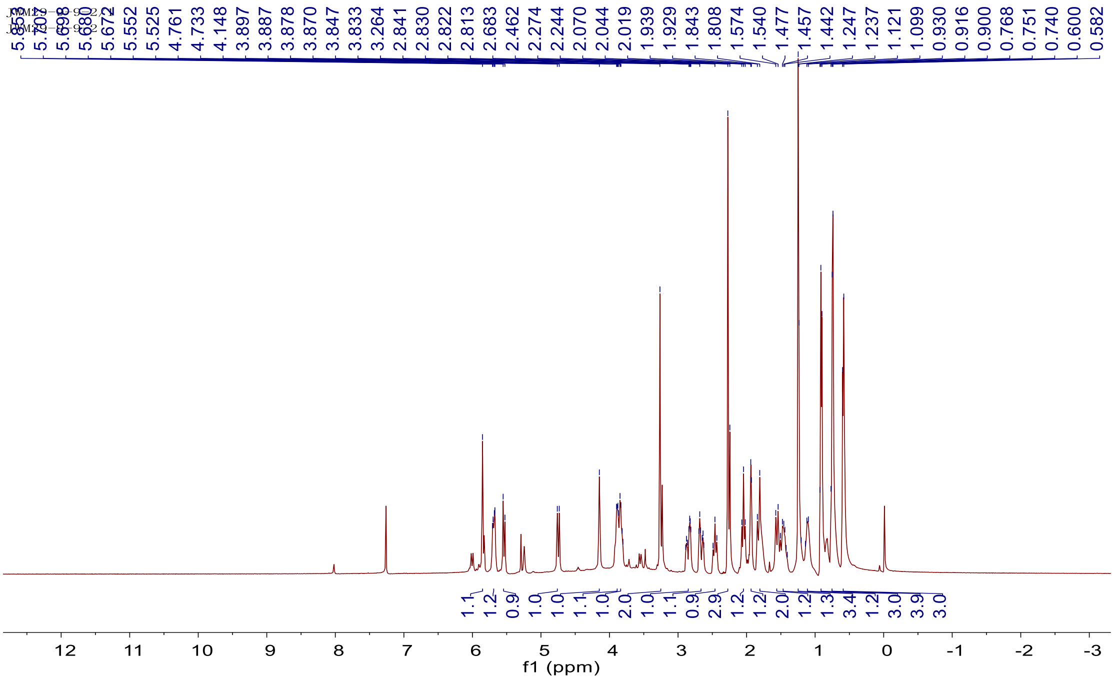

Figure S33 ^{1}H-NMR spectrum of compound **7** (in $CDCl_3$)

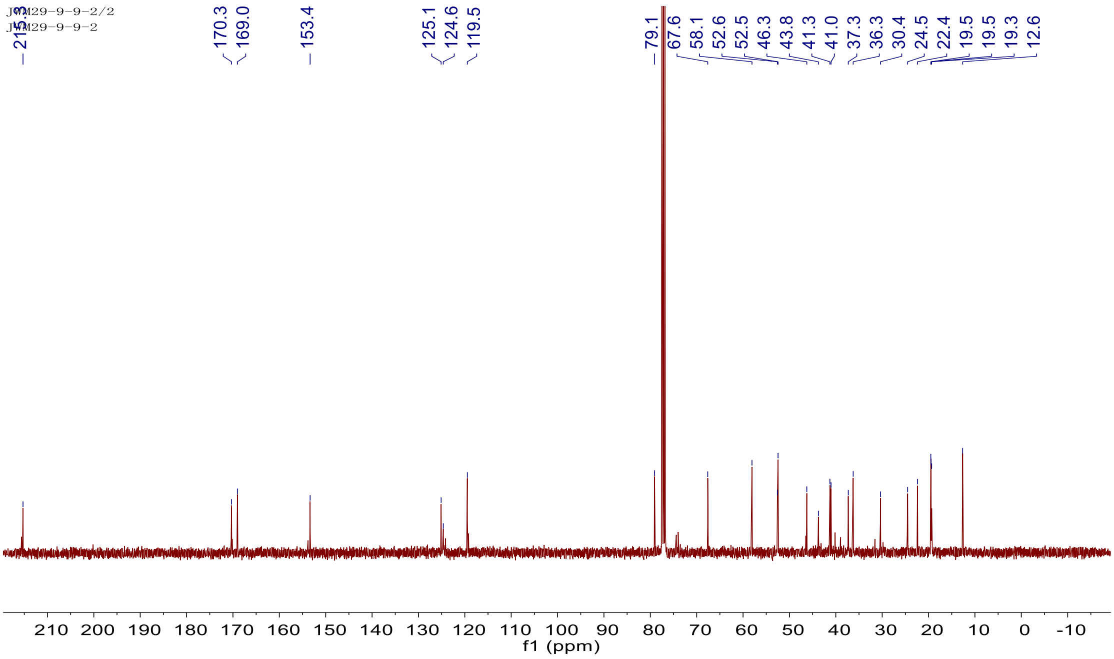

Figure S34 ^{13}C-NMR spectrum of compound **7** (in $CDCl_3$)

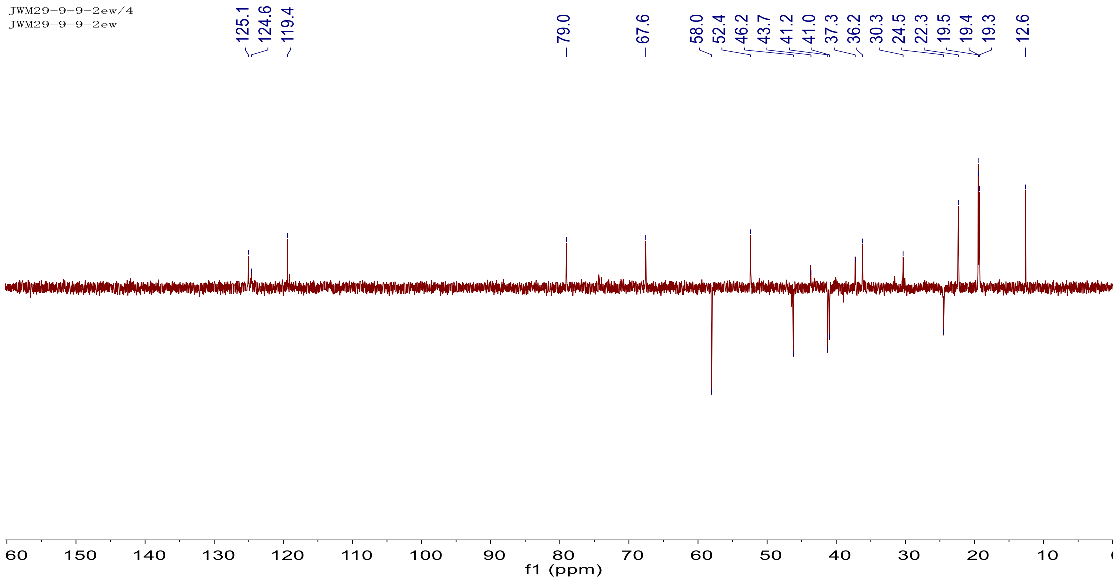

Figure S35 DEPT135 spectrum of compound **7** (in $CDCl_3$)

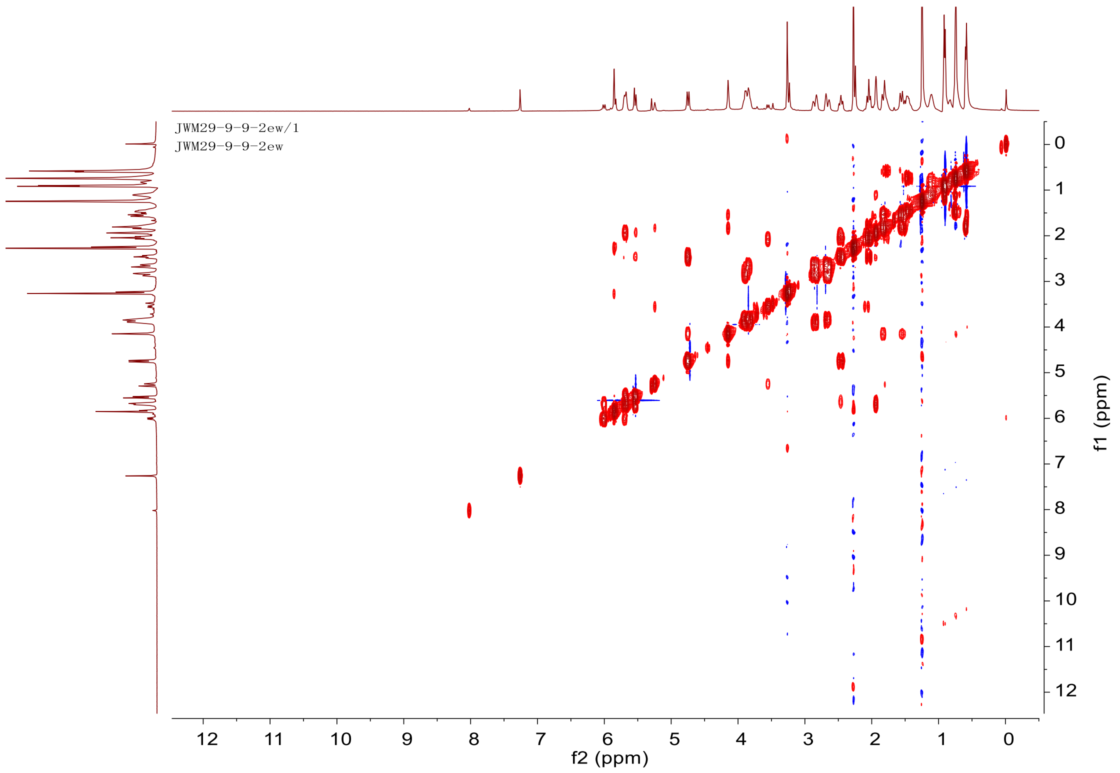

Figure S36 ^1H-^1H COSY spectrum of compound **7** (in $CDCl_3$)

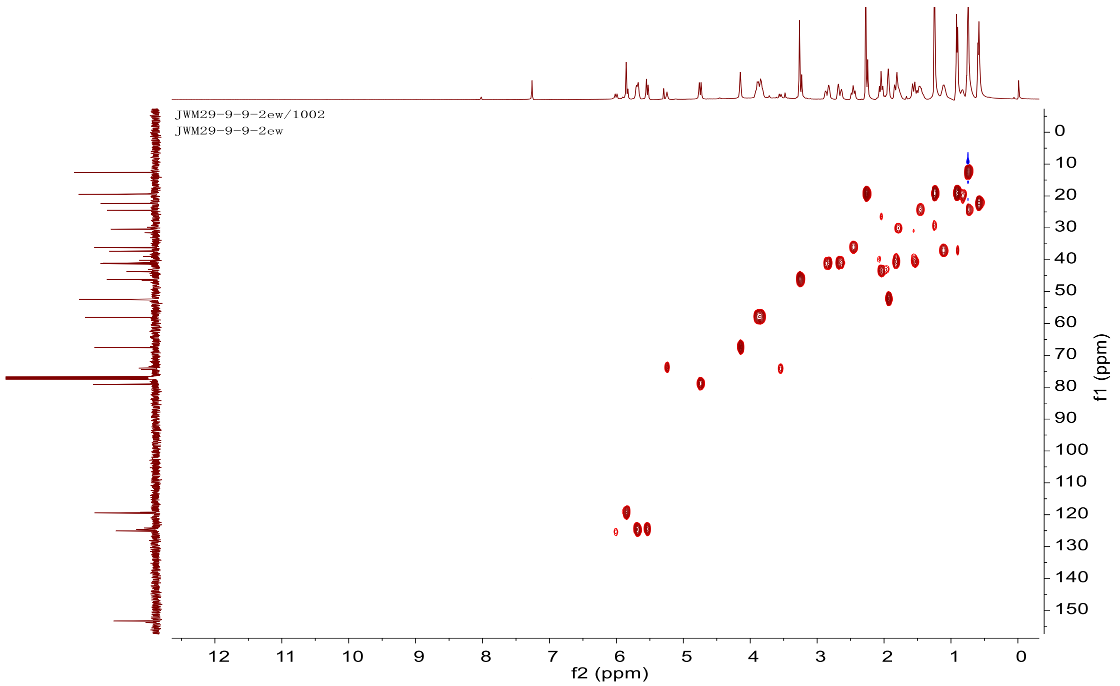

Figure S37 HSQC spectrum of compound **7** (in $CDCl_3$)

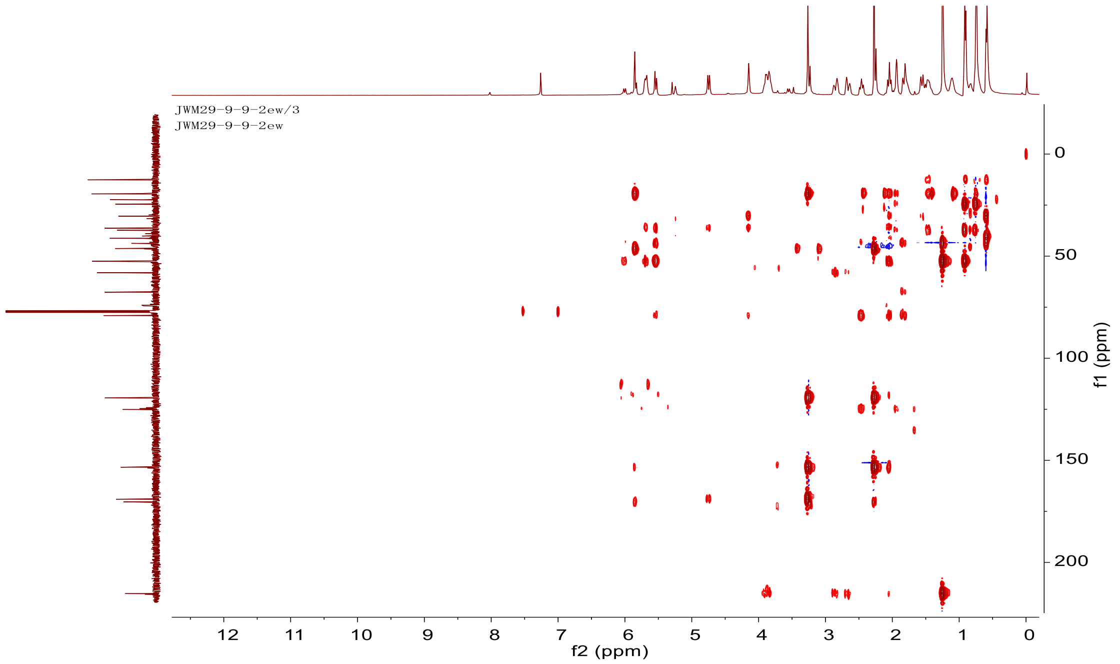

Figure S38 HMBC spectrum of compound **7** (in $CDCl_3$)

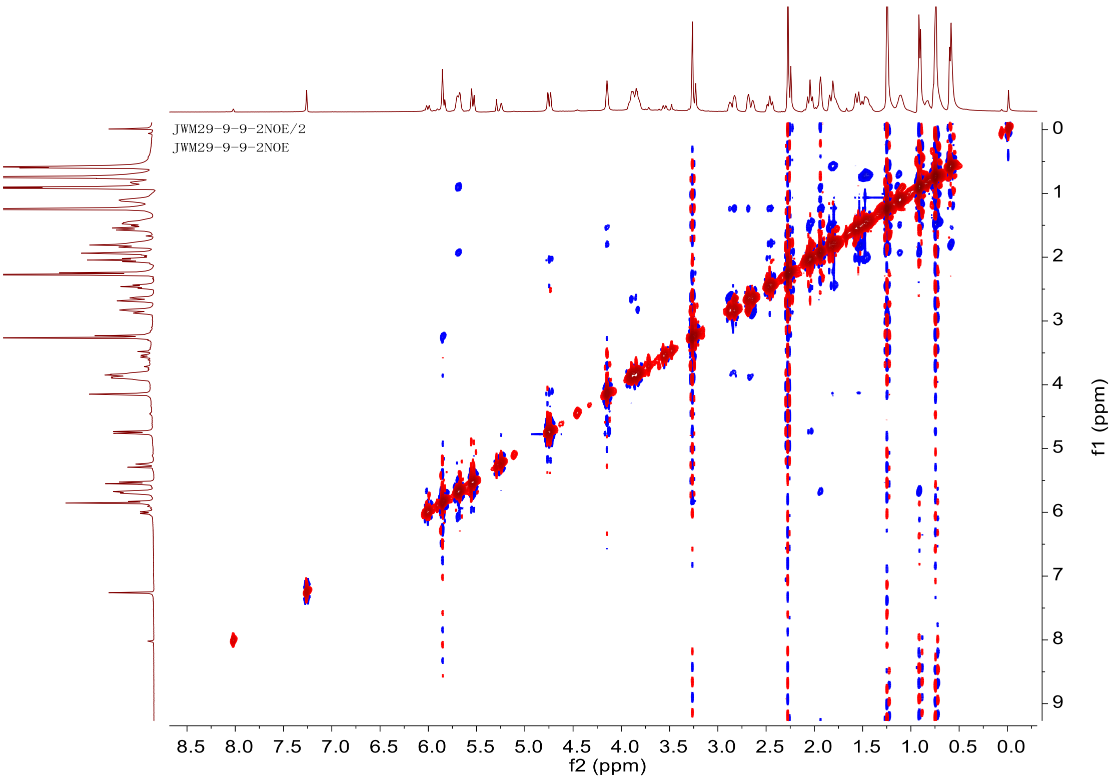

Figure S39 NOESY spectrum of compound **7** (in $CDCl_3$)

Single Mass Analysis
Tolerance = 5.0 mDa / DBE: min = -1.5, max = 50.0
Element prediction: Off
Number of isotope peaks used for i-FIT = 3

Monoisotopic Mass, Even Electron Ions
30 formula(e) evaluated with 1 results within limits (up to 50 closest results for each mass)
Elements Used:
C: 0-30 H: 0-50 O: 0-10

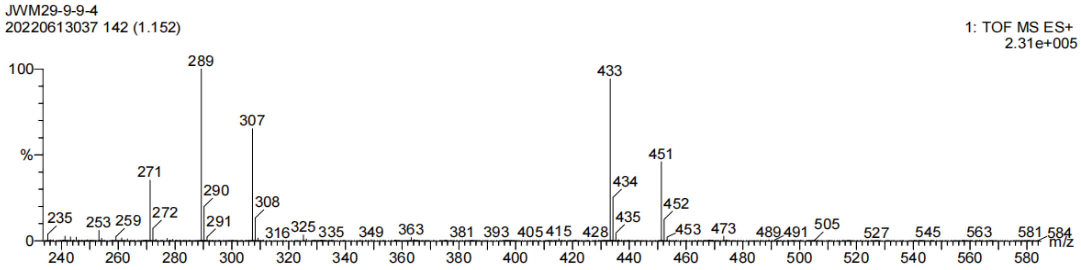

Minimum:				-1.5				
Maximum:		5.0	10.0	50.0				
Mass	Calc. Mass	mDa	PPM	DBE	i-FIT	Norm	Conf(%)	Formula
451.2697	451.2696	0.1	0.2	6.5	275.9	n/a	n/a	C25 H39 O7

Figure S40 HR-ESI-MS spectrum of compound **8**

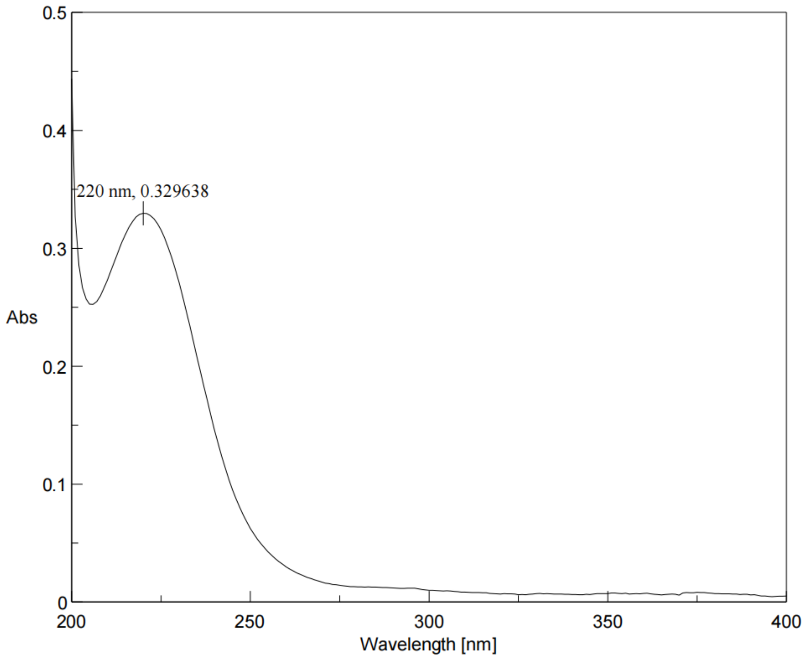

Figure S41 UV spectrum of compound **8**

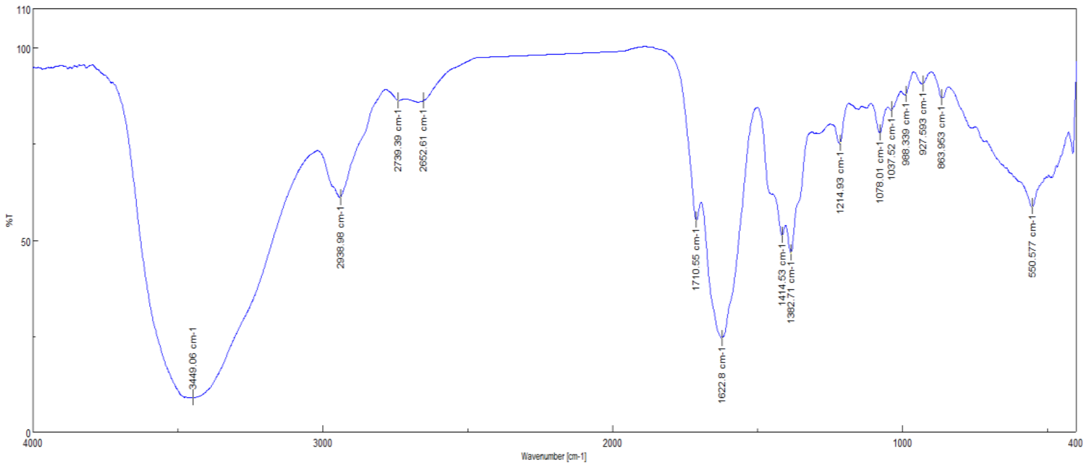

Figure S42 IR spectrum of compound **8**

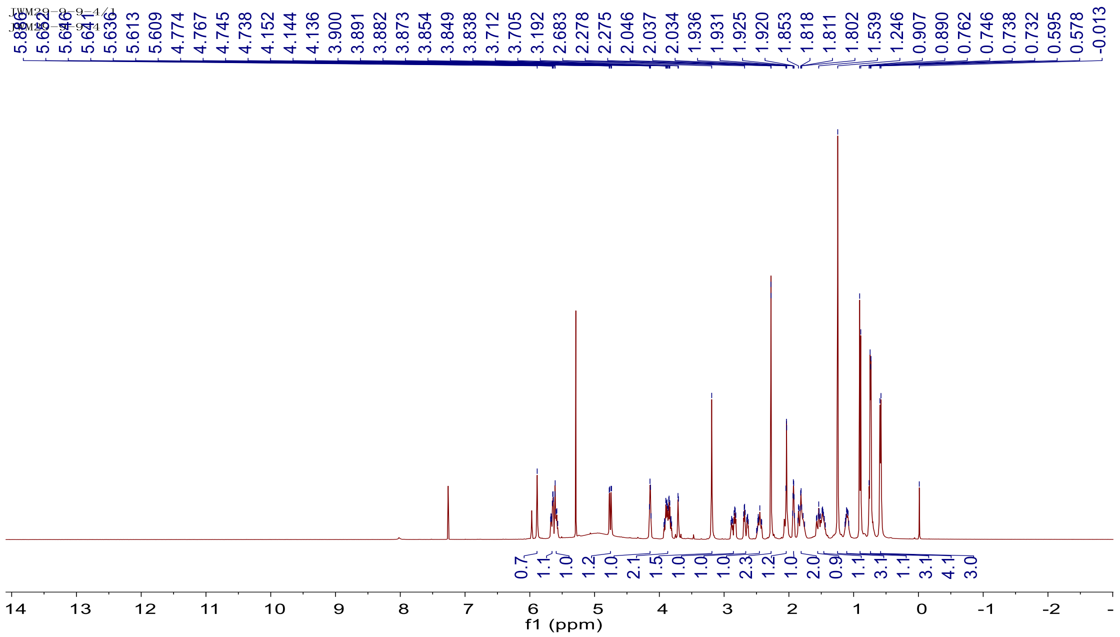

Figure S43 ^{1}H-NMR spectrum of compound **8** (in $CDCl_3$)

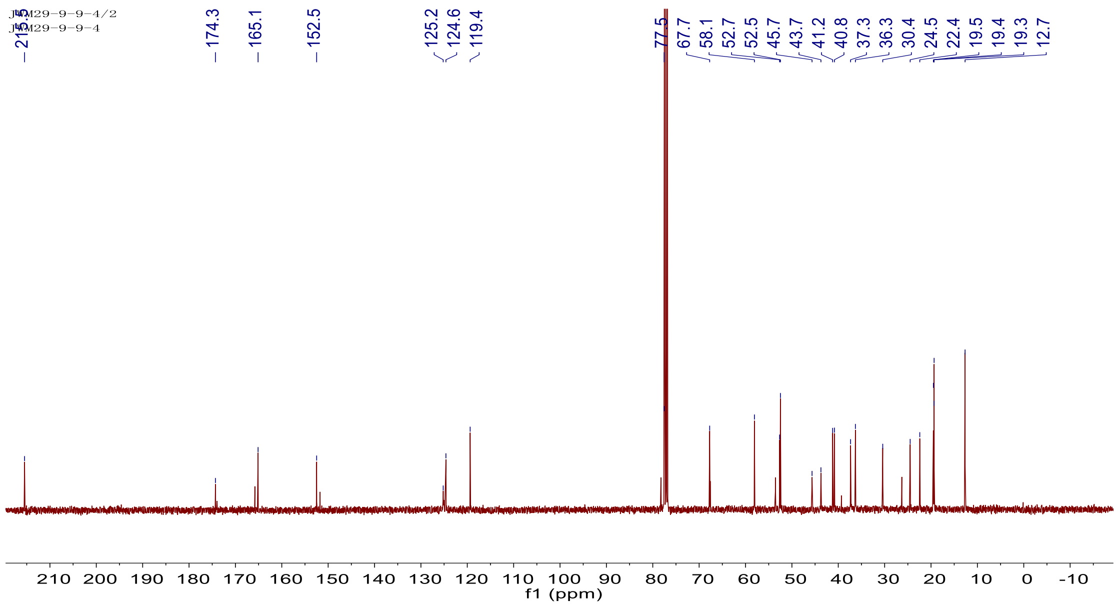

Figure S44 ^{13}C-NMR spectrum of compound **8** (in $CDCl_3$)

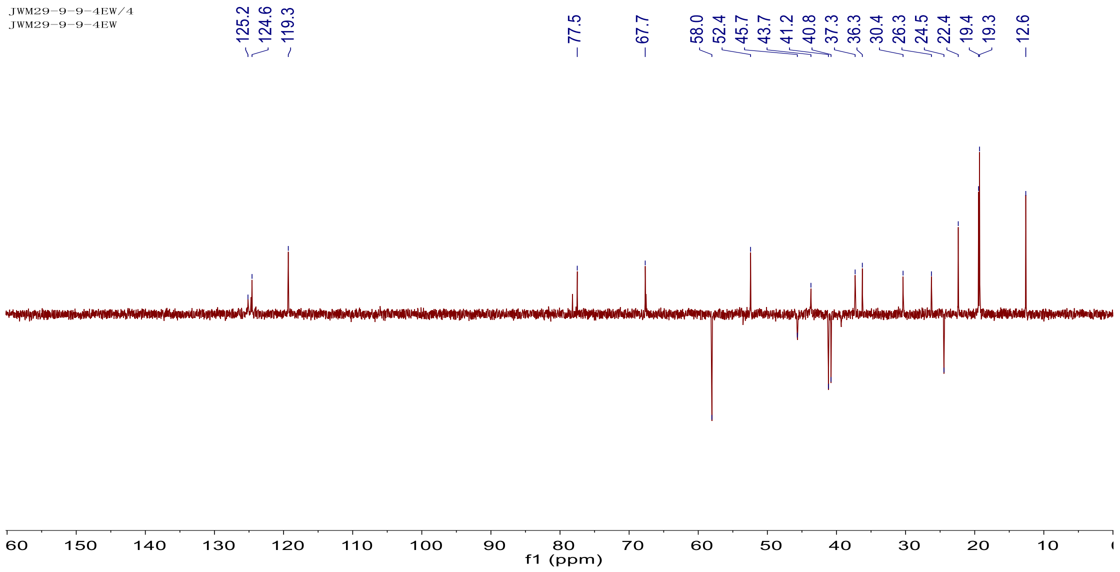

Figure S45 DEPT135 spectrum of compound **8** (in $CDCl_3$)

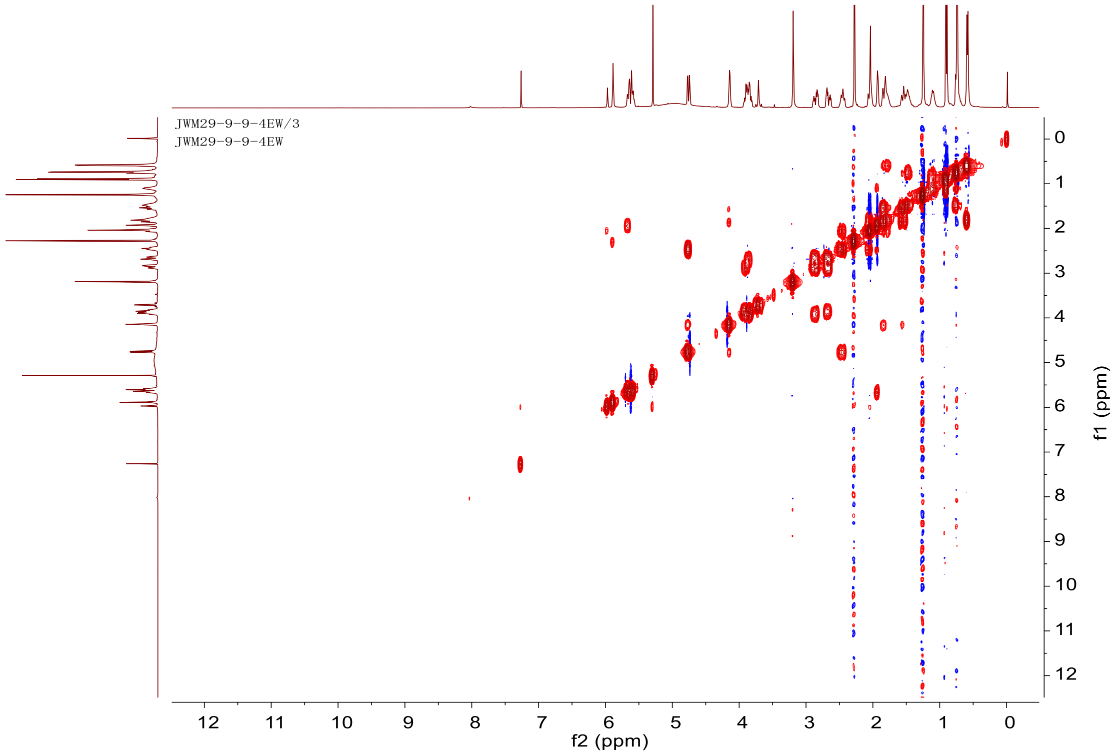

Figure S46 ^{1}H-^{1}H COSY spectrum of compound **8** (in $CDCl_3$)

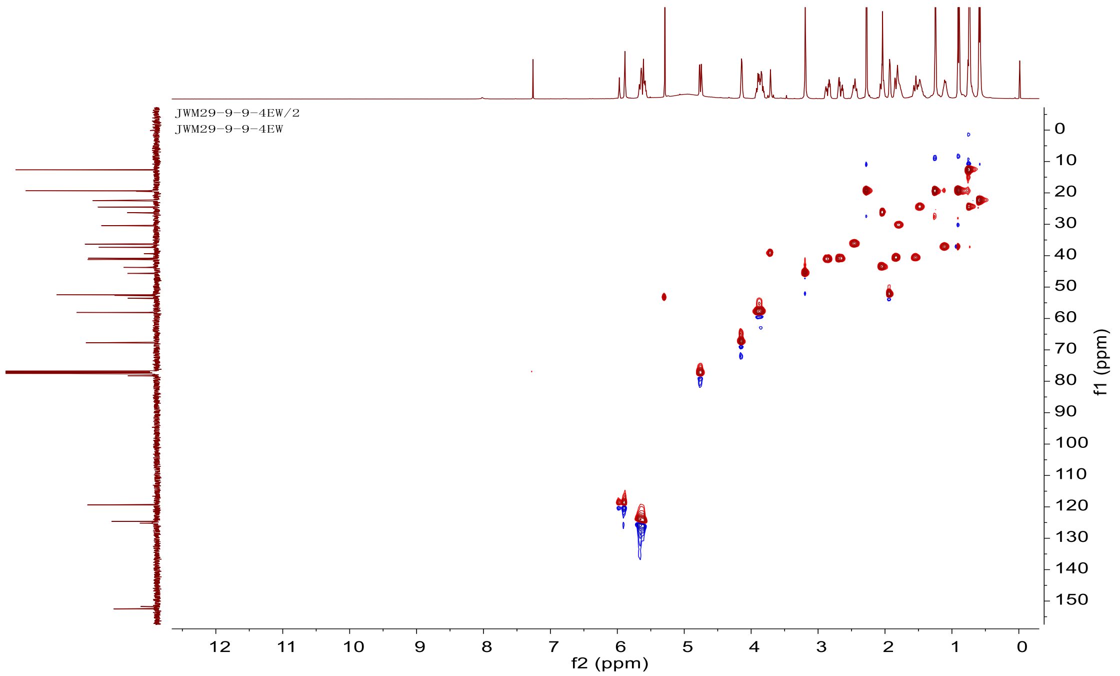

Figure S47 HSQC spectrum of compound **8** (in $CDCl_3$)

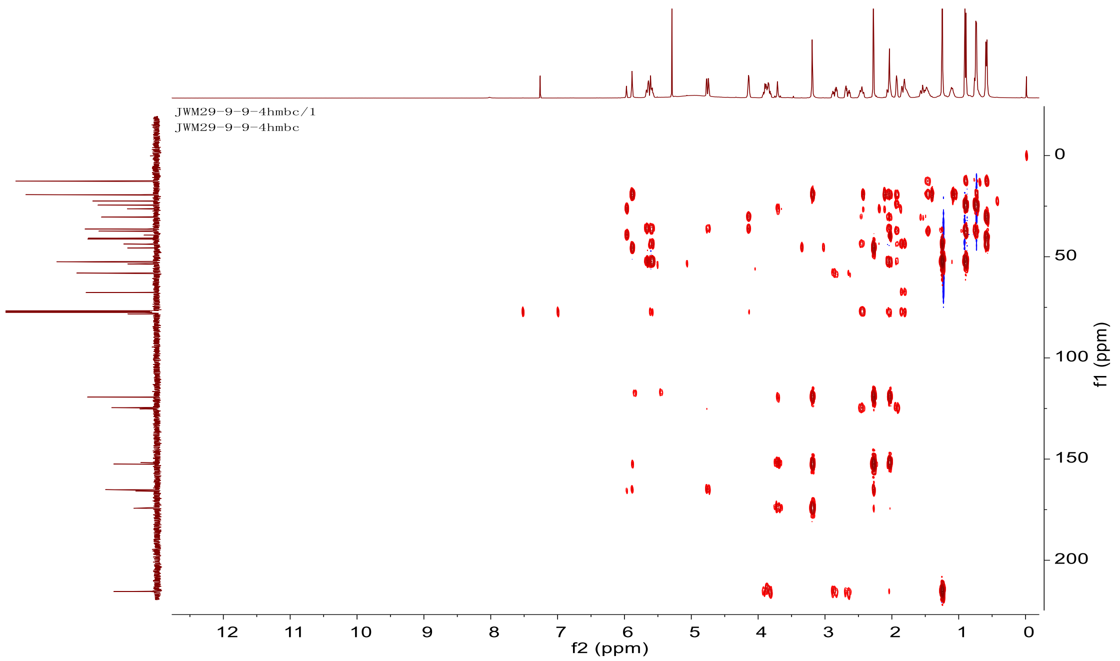

Figure S48 HMBC spectrum of compound **8** (in $CDCl_3$)

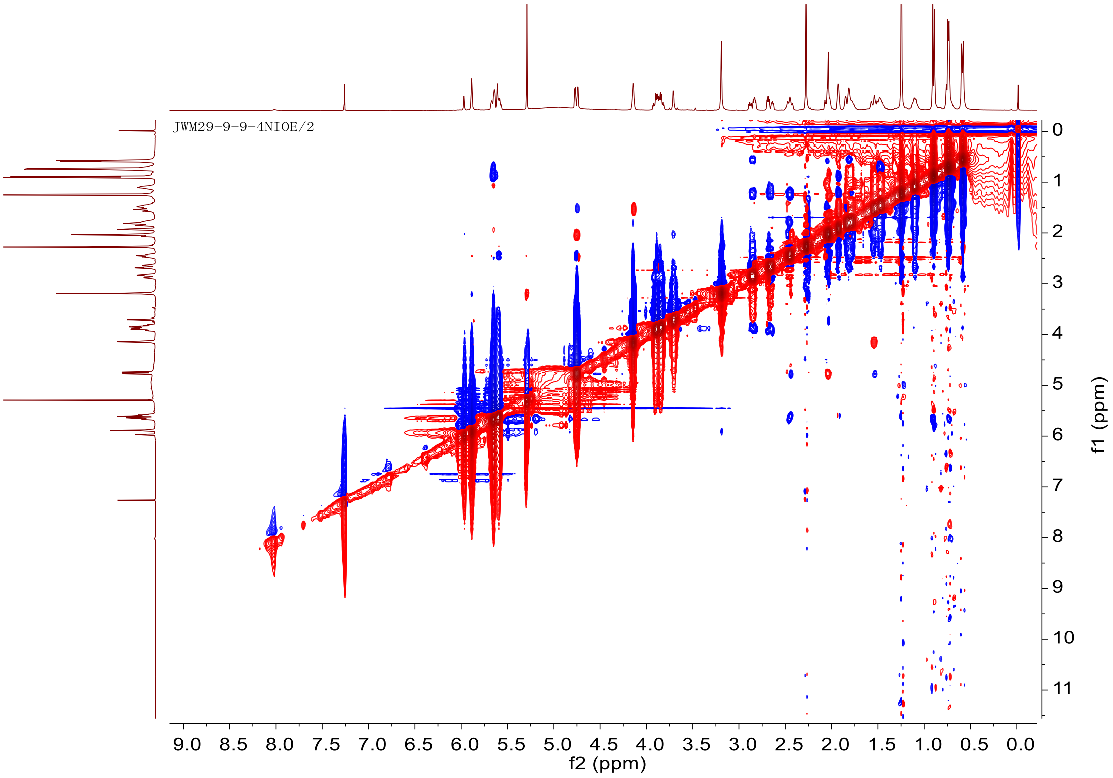

Figure S49 NOESY spectrum of compound **8** (in $CDCl_3$)

Single Mass Analysis
Tolerance = 5.0 mDa / DBE: min = -1.5, max = 50.0
Element prediction: Off
Number of isotope peaks used for i-FIT = 3

Monoisotopic Mass, Even Electron Ions
42 formula(e) evaluated with 1 results within limits (up to 50 best isotopic matches for each mass)
Elements Used:
C: 0-30 H: 0-50 O: 0-10
JWM29-9-9-2
20220613034 142 (1.152)
1: TOF MS ES+
6.34e+004

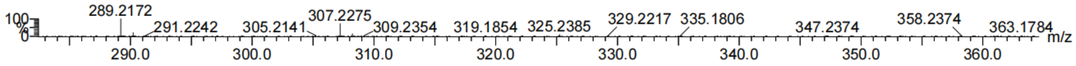

Minimum:				-1.5				
Maximum:		5.0	10.0	50.0				
Mass	Calc. Mass	mDa	PPM	DBE	i-FIT	Norm	Conf(%)	Formula
325.2385	325.2379	0.6	1.8	3.5	102.5	n/a	n/a	C19 H33 O4

Figure S50 HR-ESI-MS spectrum of compound **6A**

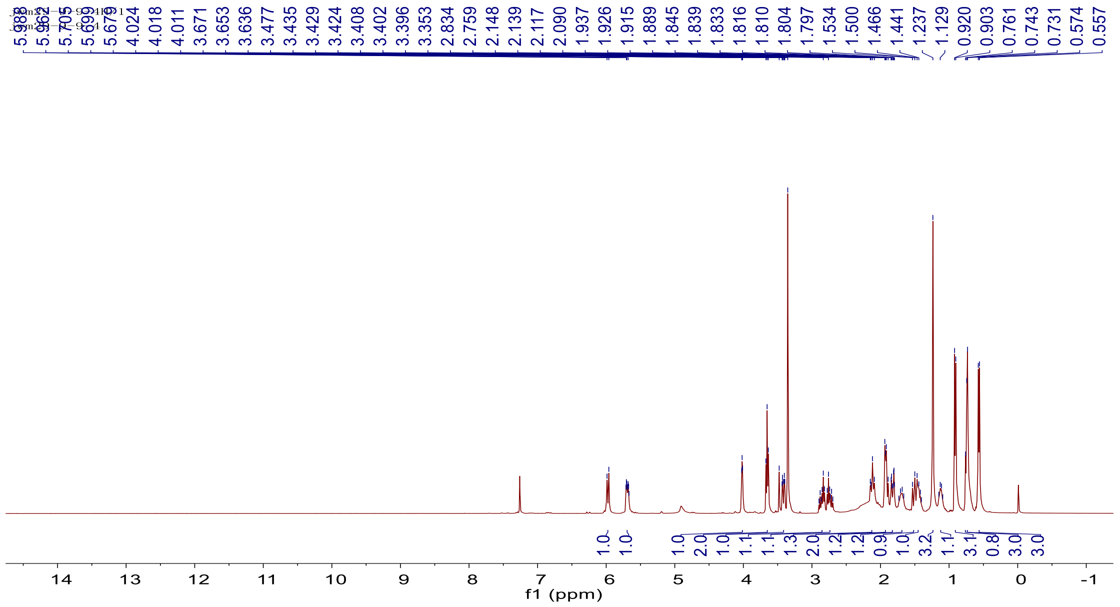

Figure S51 ^{1}H-NMR spectrum of compound **6A** (in $CDCl_3$)

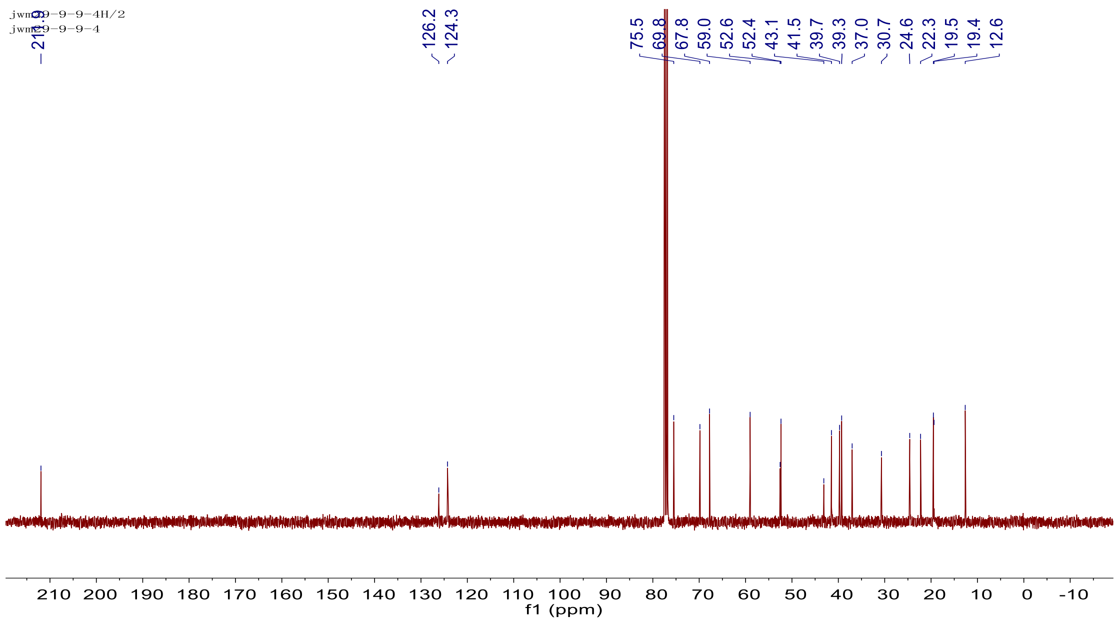

Figure S52 ^{13}C-NMR spectrum of compound **6A** (in $CDCl_3$)
